# Comparative Analysis of Physicochemical Properties, Volatile and Metabolite Profile of Lotus Sprouts from Varying Cultivars and Grades

**DOI:** 10.3390/foods15213965

**Published:** 2026-11-04

**Authors:** Jiao Liu, Jingxuan Li, Yanyi Li, Yang Xu, Hua Ding, Xun Zhang, Yijie Wu, Jie Yang, Youxiang Zhou

**Affiliations:** 1 Hubei Key Laboratory of Nutritional Quality and Safety of Agro-Products, Institute of Quality Standard and Testing Technology for Agro-Products, Hubei Academy of Agricultural Sciences, Wuhan 430064, China; babojiao@126.com (J.L.); xuyang@hbaas.ac.cn (Y.X.);; 2 Key Laboratory of Cold Chain Logistics Technology for Agro-Product, Ministry of Agriculture and Rural Affairs, Wuhan 430064, China

**Keywords:** lotus sprout, cultivar, part, volatile aroma compound, metabolomics, alkaloid, comparative analysis

## Abstract

Lotus sprouts are a widely consumed aquatic vegetable, yet their quality remains poorly characterized. This study integrated physicochemical analysis, volatile flavor profiling, and metabolomics to evaluate quality differences among three lotus sprout cultivars, as well as different parts and grades. The apical portions had significantly higher contents of protein, total phenolics, and γ-aminobutyric acid than the stem portions. A total of 46 volatile compounds were identified by headspace solid-phase micro-extraction combined with gas chromatography–mass spectrophotometry (HS-SPME-GC-MS), and the abundance of 2,4-di-tert-butylphenol and 1-octen-3-ol was further confirmed using standards. Untargeted metabolomics identified 335 compounds, with sugars, polyphenols, and amino acids being the most abundant categories. Differentially accumulated metabolites between parts and cultivars were significantly enriched in glutathione metabolism, flavonoid biosynthesis, and amino acid metabolism. Notably, the two Baiyuzan cultivars generally accumulated higher levels of beneficial metabolites than Wuzhi No. 2, whereas the stem portion of the higher-grade Wuzhi No. 2 showed lower accumulation of sucrose and paprazine. Quantitative analysis of eight alkaloids using external standard calibration curves indicated that nuciferine was the most abundant alkaloid (13.3 to 31.1 μg/g). Collectively, this study provides a foundational framework for evaluating the metabolite profiles and application potential of lotus sprouts.

## 1. Introduction

Lotus (*Nelumbo nucifera* Gaertn.) is a famous perennial aquatic plant with a cultivation history of over 3000 years in China and Southeast Asia [1]. Nearly all parts of the lotus plant, including leaves, flowers, seeds, and rhizomes, are utilized across various industries such as food, medicine, and cosmetics [2,3,4]. Among them, lotus sprout refers to the tender rhizomes in the early growth stage of the lotus rhizome. Lotus sprout is characterized by its slender tubular shape has gradually attracted attention due to its crisp texture, refreshing flavor, and high nutritional value [5]. In China, the lotus sprout harvesting period typically spans from May to August each year [6,7]. As a distinct seasonal product, lotus sprouts meet consumer demand during the period before mature lotus rhizomes become available in the market.

Lotus sprouts are usually priced 4 to 10 times higher than mature lotus rhizome [8]. Top-grade commercial sprouts have a pen-tip-shaped apical part without any side buds or branches. Based on the degree of sprouting of these tips, lotus sprouts can be classified into three grades; the larger the lateral buds or branches, the lower the grade and the lower the price. Lotus sprouts are mainly harvested in the hot and humid summer conditions, and are prone to discoloration and even blackening, severely affecting their market value and consumer acceptance. Therefore, existing research has mainly focused on proper preservation and processing methods to extend the shelf life of lotus sprouts and their derived products [8,9]. As a relatively new type of aquatic vegetable, limited information is available regarding the nutritional differences of lotus sprouts caused by cultivar, cultivation environment, harvesting time, and storage conditions.

Multi-omics approaches have been widely applied to characterize the nutritional composition, volatile profiles, and metabolite landscapes of foods and agricultural products [10,11]. These methods can comprehensively reveal differences among cultivars, processing methods, and growth environments [12,13]. Lotus sprouts contain various bioactive compounds, including alkaloids and volatile flavor substances, which contribute to their unique taste and aroma and may also offer potential health benefits [14]. Nevertheless, an in-depth understanding of the aroma and metabolite profiles of lotus sprouts remains lacking. Elucidating the composition and content of functional metabolites in lotus sprouts will help to explore their edible and medicinal value and provide a theoretical basis for the deep processing and utilization of lotus sprout resources.

In this study, three common lotus sprout cultivars—Wuzhi No. 2, Baiyuzan No. 1, and Baiyuzan No. 3—were collected to comprehensively assess the variations in basic nutritional components, bioactive substances, volatile flavor compounds, and metabolic profiles. Comparisons were made both among cultivars and among different commercial grades within the same cultivar. These findings are expected to provide a fundamental data reference for the future development and utilization of lotus sprout resources and for the improvement of their edible quality.

## 2. Materials and Methods

### 2.1. Chemicals and Reagents

Chemical standards of nuciferine (purity ≥ 98%), liensinine (purity ≥ 98%), neferin (purity ≥ 98%), dehydronuciferine (purity ≥ 98%), O-nornuciferine (purity ≥ 95%), N-nornuciferine (purity ≥ 98%), roemerine (purity ≥ 98%), asimilobine (purity ≥ 98%), 2,4-di-tert-butylphenol (purity ≥ 98%), benzothiazole (purity ≥ 96%), hexadecane (purity ≥ 98%) and 1-octen-3-ol (purity ≥ 98%) were purchased from Shanghai yuanye Bio-Technology Co., Ltd. (Shanghai, China). LC-MS-grade acetonitrile and formic acid were obtained from Sigma-Aldrich (St. Louis, MO, USA).

### 2.2. Sample Collection and Pretreatment

Three lotus sprout cultivars, Wuzhi No. 2, Baiyuzan No. 1 and Baiyuzan No. 3, were harvested in July 2024 from the same planting base at Honghu City, Hubei Province. For each cultivar, three independent samples were collected from separate sampling points. All samples were packed individually in bags with ice, and immediately transported to the laboratory. After the surface moisture was wiped off, the Wuzhi No. 2 samples were graded according to the morphology of the apical part. For Baiyuzan No. 1 and No. 3, only first-class samples were retained. The apical and stem sections of each sample were separately collected, freeze-dried, ground into powder, and stored at −80 °C. Details of the samples are presented in Table 1.

### 2.3. Determination of Protein, Free Amino Acid and Sugar Contents

The crude protein content was determined using the Kjeldahl method, with a conversion factor of 6.25. A 0.4 g sample was weighed and mixed with 6 mL of 0.02 mol/L hydrochloric acid. The mixture was then subjected to ultrasonic extraction in a 95 °C water bath for 20 min, followed by centrifugation at 5000 r/min for 15 min. The supernatant was collected and diluted to a final volume of 10 mL. The solution was aspirated, filtered through a 0.22 μm membrane filter, and transferred to a sample vial for free amino acid (FAA) analysis by using an automatic amino-acid analyzer (L-8900; Hitachi Ltd., Tokyo, Japan). The amount of each amino acid present in the sample was calculated using a standard sample [15]. The soluble sugar and reducing sugar contents were determined using the 3,5-dinitrosalicylic acid (DNS) method. Each sample was analyzed in triplicate.

### 2.4. Determination of Total Phenolics

The total phenolic content (TPC) was determined with reference to the Folin–Ciocalteu method. A stock standard solution of gallic acid at a concentration of 250 μg/mL was prepared. Prior to use, standard working solutions with mass concentrations of 0, 15, 25, 50, 75, 100, and 125 μg/mL were freshly prepared to construct the standard curve. A 0.2 g sample was weighed and extracted by shaking with either 5 mL of distilled water (marked as TPC-W) or 5 mL of methanol for 30 min (marked as TPC-M). The mixture was then centrifuged at 5000 r/min for 15 min at 4 °C, and the supernatant was collected. The extraction process was repeated twice. The combined supernatants were evaporated to near dryness at 45 °C, followed by reconstitution with 5 mL of distilled water. The absorbance was measured at a wavelength of 725 nm. The total phenolic content in each sample was calculated using the standard curve (y = 0.0044x + 0.0013, R^2^ = 0.9996) and expressed as the amount of gallic acid equivalent per gram of sample (mg/g). Each sample was analyzed in triplicate.

### 2.5. Analysis and Validation of Volatile Flavor Compounds by HS-SPME GC-MS

The volatile flavor compounds profile of lotus sprout were analyzed by HS-SPME combined with GC-MS (Agilent 8890-7000E, Agilent, Santa Clara, CA, USA). For compound extraction, 0.5 g freeze-dried lotus sprout sample was weighed and placed into a 20 mL headspace vial. Add 10 mL of distilled water and vortex to ensure thorough mixing. Then add 5 μL of ethyl decanoate (62.5 μg/mL) as an internal standard. Promptly seal the vial tightly and place it on the sample tray for extraction by the SPME autosampler (PAL RSI 120, CTC Analytics, Zwingen, Switzerland) in headspace solid-phase microextraction mode [16]. The automated extraction conditions for the sample were as follows: incubated at 60 °C for 20 min; extraction temperature, 60 °C; extraction time, 30 min; and sample desorption time, 3 min.

The chromatographic conditions were set as follows: injector port temperature, 250 °C; carrier gas, helium (99.999% purity); flow rate, 1.2 mL/min; column, Agilent DB-5MS (30 m × 0.25 mm, 0.25 μm film thickness). The column temperature was programmed as follows: the initial temperature was set at 50 °C and held for 2 min, then increased to 80 °C at a rate of 5 °C/min and held for 4 min, followed by a further increase to 250 °C at a rate of 4 °C/min and held for 1 min. The injection volume was 1 μL, with a splitless injection mode.

The mass spectrometry conditions were set as follows: ion source and detector voltages set at 70 eV and 1372 V, respectively; quadrupole temperature, 150 °C; ion source temperature, 280 °C; mass range, 50–550 amu; full scan mode employed. The mass spectrometry data were initially identified by comparison with the NIST2014 database for volatile components. Spectral interpretation was further conducted in conjunction with relevant literature to achieve qualitative analysis of the volatiles. Chromatographic peaks with a similarity index ≥700 and appearing in at least two out of three replicate samples were selected as components present in the sample.

The volatile compounds tentatively identified by mass spectral library matching were semi-quantified using the internal standard method [17]. The calculation formula was as follows: $C\;=\;(A/A_{i\;})\;\times \;C_{i}$, where *A* and *A_i_* are the peak areas of the compound and internal standard, and *C* and *C_i_* are the concentrations of the compound and internal standard in the sample.

Subsequently, four representative compounds, hexadecane, 1-octen-3-ol, 2,4-di-tert-butylphenol, and benzothiazole, were analyzed using external standard calibration curves to further support compound identification and estimate concentration trends. The standard calibration curves and characteristic ion parameters were summarized in Table S1. For the flavor compound extraction, 0.25 g freeze-dried lotus sprout sample was mixed with 4 mL n-hexane and 4 mL saturated salt water. The mixture was vortexed for 30 s and then subjected to ultrasonic treatment for 5 min. After standing for 10 min, the supernatant was collected and adjusted to a final volume of 5 mL. A total of 2 μL internal standard solution (ethyl decanoate, 12.5 μg/mL) was added, and the mixture was filtered before instrumental detection. The chromatographic and mass spectrometric conditions were consistent with those described above, and quantification was performed under selected ion monitoring mode.

### 2.6. Non-Targeted Metabolomics Analysis by UPLC-Q-TOF-MS

A total of 40 mg freeze-dried lotus sprout sample was accurately weighed and mixed with 2 mL pre-cooled 50% (*v*/*v*) methanol. The mixture was homogenized and then subjected to ice-bath ultrasonication for 30 min. After extraction, the sample was centrifuged at 12,000 rpm for 10 min at 4 °C. The supernatant was collected and filtered through a 0.22 μm organic membrane before LC-MS injection. The mass spectrometric data of all samples were acquired with an information-dependent acquisition (IDA) method using the ZenoTOF 7600 mass spectrometer (SCIEX, Framingham, MA, USA) in electrospray ionization (ESI) positive and negative ion modes. The pretreatment, mass spectrometric data acquisition, and data processing were all conducted based on the method we had established previously [13]. Briefly, chromatographic separation was achieved on a SCIEX ExionLC AD system equipped with an ACQUITY UPLC HSS T3 column (2.1 × 100 mm, 1.8 µm) maintained at 40 °C. A gradient elution program consisting of mobile phases A (0.05% formic acid in water) and B (acetonitrile) was used at a flow rate of 0.4 mL/min, and the gradient program was as follows: 0–1 min, 1% B; 1–24 min, 1–30% B; 24–37 min, 30–98% B; 37–42 min, 98%; 42.1–44 min, 1% B. The injection volume was 5 µL, and the sample plate was maintained at 4 °C. Each sample was analyzed in triplicate. The raw mass spectral data were acquired in TOFMS-IDA-TOFMSMS mode and analyzed using SCIEX OS software (version 2.2, AB SCIEX, Framingham, MA, USA). Compound identification was performed by using the SCIEX self-constructed metabolite standard spectrum database (OS Database). The analysis employed stringent parameters, including a mass deviation threshold of <5 ppm and a signal intensity deviation of 30%. The metabolite identification levels were classified into four tiers: Level 1: The parent ion, fragment ions, and retention time all align with the identification results; Level 2: The parent ion and secondary mass spectrum fragment ions match the identification results, with a library score of ≥60; Level 3: The parent ion and fragment ions match the identification results, but the library score is <60; Level 4: Only the parent ion corresponds to the identification results.

### 2.7. Extraction of Alkaloids

The extraction of alkaloids was based on the reference method with some modification [18]. A total of 0.5 g of lotus sprout sample was weighed and mixed 15 mL of petroleum ether for defatting. After removing the petroleum ether, add 10 mL of 65% ethanol and soak for 1 h, and then perform ultrasonic extraction at 30 °C for 40 min. The mixture was centrifuged at 5000 rpm for 3 min, and the supernatant was collected and filtered. Then, 5 mL of the filtrate was evaporated to dryness in a water bath, and the residue was redissolved in a 5% hydrochloric acid solution. Subsequently, an equal volume of chloroform was added for three rounds of extraction. The combined chloroform layers were collected, evaporated to dryness and the residue was dissolved in anhydrous ethanol. After filtration, the solution was adjusted to 5 mL by volumetric flask. The resulting alkaloid extract was stored at −20 °C for future use.

### 2.8. Detection of Alkaloids by UPLC-MS/MS

Analysis of alkaloids was carried out using an Acquity H-Class UPLC system equipped with a Waters Xevo TQ-S Micro MS system (Waters, Milford, MA, USA). An ACQUITY UPLC HSS T3 column (100 mm × 2.1 mm, 1.8 µm) was sustained at 40 °C to achieve the chromatographic separation. The mobile phase comprised Mobile Phase A (0.1% formic acid in water) and Mobile Phase B (acetonitrile), and the flow rate was maintained at 0.3 mL/min. The injection volume was 1 μL, and gradient elution was carried out. It started with 90% Mobile Phase A, decreased to 15% Mobile Phase A from 0.3 to 3.5 min, followed by a 2 min hold time. Then, it reverted to the initial 90% Mobile Phase A, with a 2 min equilibration time between runs.

The mass spectrometer was employed with a positive mode maintained for the ESI ion source. The ion source and desolvation temperatures were maintained at 150 °C and 450 °C, respectively. Capillary voltage was set to 2.0 kV, and the desolvation gas flow was 1000 L/h. Multiple reaction monitoring (MRM) mode was used for data acquisition. MRM conditions were tabulated in Table S2. Quantification of certain alkaloids in lotus sprouts was performed using standard calibration curves (Table S3).

### 2.9. Statistical Analysis

Statistical data were expressed as means ± standard deviation (*n* = 3). Significance was assessed by one-way analysis of variance (ANOVA) using SPSS 16.0. The presence of distinct lowercase letters indicates statistically significant differences (*p* < 0.05). Principal component analysis (PCA) was performed by statistics function prcomp within R (www.r-project.org, accessed on 28 August 2025). The data was unit variance scaled before unsupervised PCA. For two-group analysis, differential accumulation metabolites (DAMs) were determined by Variable Importance in Projection (VIP, VIP > 1), absolute Log_2_FC (|Log_2_FC| ≥ 1.0) and FDR (adjusted *p*-value) < 0.05. VIP values were extracted from the Orthogonal Partial Least Squares Discriminant Analysis (OPLS-DA) result, which also contain score plots and permutation plots, and was generated using the R package MetaboAnalystR. The data were log-transformed (Log_2_) and mean-centered before OPLS-DA. In order to avoid overfitting, a permutation test (200 permutations) was performed. Identified metabolites were annotated using the KEGG Compound database (http://www.kegg.jp/kegg/compound/, accessed on 28 August 2025), annotated metabolites were then mapped to the KEGG Pathway database (http://www.kegg.jp/kegg/pathway.html, accessed on 28 August 2025). Based on the DAMs, KEGG pathway enrichment analysis was performed. The Rich Factor was calculated as the ratio of the number of DAMs in a given pathway to the total number of metabolites annotated to that pathway, with a larger value indicating a higher degree of enrichment. Pathways with *p*-value < 0.05 were defined as significantly enriched pathways for DAMs. Bubble plots were generated for the top 20 pathways with the smallest *p*-values.

## 3. Results

### 3.1. Comparison of Basic Nutritional Quality

The basic nutritional components, including moisture, protein, free amino acid (FAA), sugar, and total phenolics, of different parts of lotus sprouts were compared (Table 2). Generally, the moisture content of all samples was above 93%. Comparisons of relevant nutritional indicators were conducted based on dry-basis results. The analytical results indicated that lotus sprouts primarily contained reducing sugars. The sugar content in the apical section of lotus sprouts was approximately half that of the stem section. On the contrary, the content of other nutrients in the apical portion of lotus sprouts was significantly higher than that in the stem portion. For example, the protein content in the apical portion was 1.4 to 1.9 times that in the stem, and the TPC extracted with water from the apical portion was 1.2 to 1.8 times that of the stem. In addition, extraction with methanol was more conducive to the release of phenolic compounds. In particular, the TPC in the stem increased by 1.3–1.7-fold. A total of 14 amino acids were detected, and γ-aminobutyric acid (GABA) was the most abundant component, accounting for 22–33% of the total free amino acid content.

### 3.2. Profile of Volatile Flavor Compounds

**Figure 1:**
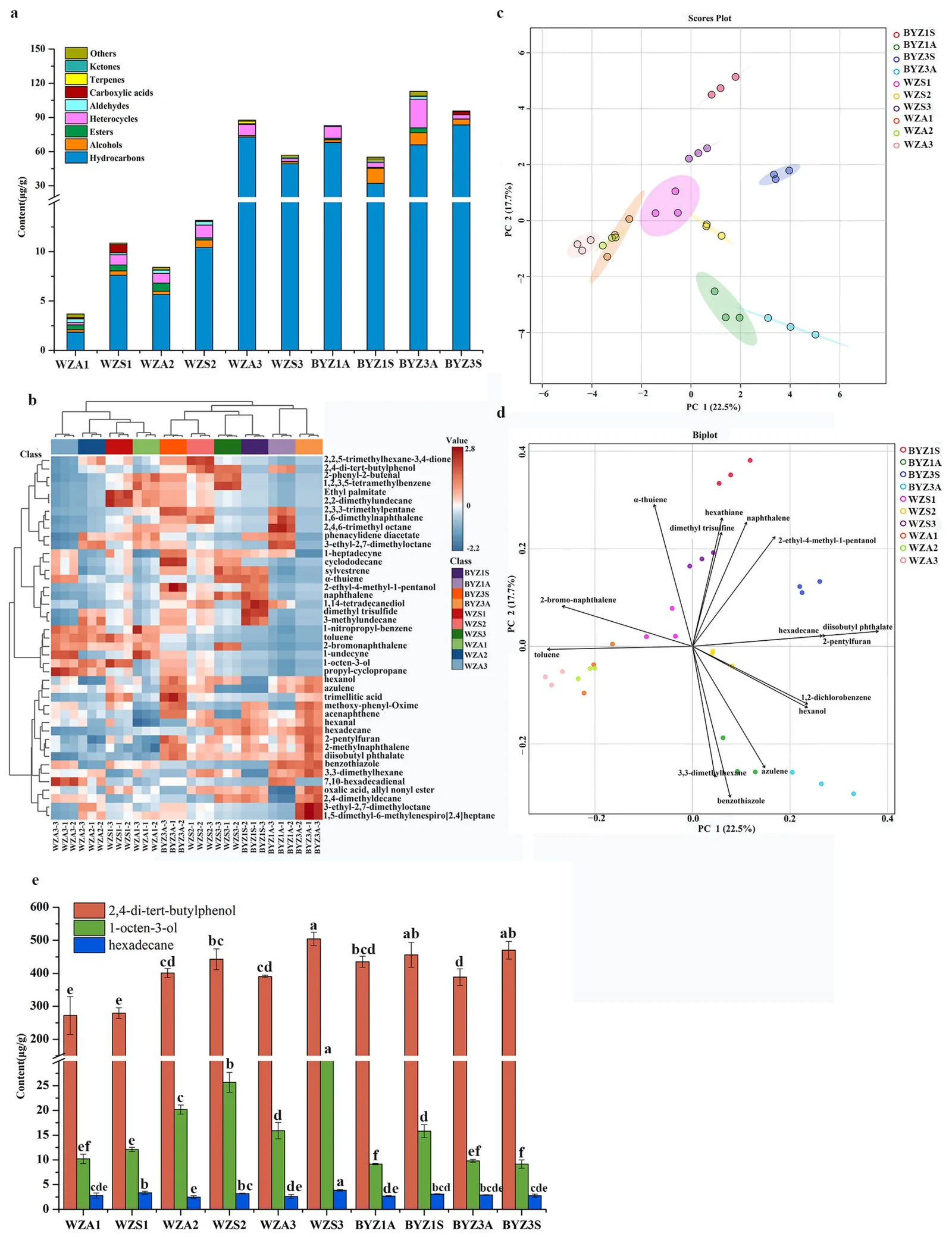
Volatile flavor compounds analysis of different cultivars and parts of lotus sprout. (**a**) Statistical map of different compound types. (**b**) Heat map analysis. PCA score plot (**c**) and biplot (**d**) of each comparison group. Each circle represents an individual sample, with different colors indicating different groups as detailed in the legend. The colored shaded ellipses represent 95% confidence intervals, illustrating the variance and distribution of samples within each group. (**e**) Quantitative determination of characteristic flavor compounds. Data are presented as mean ± standard deviation (SD) (*n* = 3), different lowercase letters indicate significant differences between groups (*p* < 0.05). WZA1 and WZS1 represent the apical and stem sections of Wuzhi No. 2 at Grade 1, respectively; WZA2 and WZS2 correspond to Grade 2, and WZA3 and WZS3 correspond to Grade 3. BYZ1A and BYZ1S are the apical and stem sections of Baiyuzan No. 1 at Grade 1, and BYZ3A and BYZ3S are those of Baiyuzan No. 3 at Grade 1.

The volatile flavor compounds in ten groups of lotus sprouts were analyzed using HS-SPME-GCMS. A total of 46 volatile compounds were detected across all samples, including 22 hydrocarbons, 5 alcohols, 5 heterocycles, 3 esters, 2 aldehydes, 2 carboxylic acids, 2 terpenes, 1 ketone, and 4 other substances (Table S4). To understand the distribution of volatile flavor compounds among lotus sprout cultivars, the volatile compounds were semi-quantified using the internal standard method, and the results were expressed as relative concentration. As showed in Figure 1a, the most abundant volatile compounds in all samples were hydrocarbons. The contents of ester compounds and all volatile compounds in Baiyuzan No. 1 and Baiyuzan No. 3 were both relatively higher than those in Wuzhi No. 2. Specifically, the ester content in the apical part of BYZ No.3 (BYZ3A) reached 4.28 μg/g, which was 3.6 and 8.7 times that of BYZ1A and WZA1, respectively. Similarly, the total content of the 46 compounds in BYZ1A and BYZ3A reached 112.96 and 82.82 μg/g, which were 22.5 times and 30.7 times that of WZA1, respectively. The contents in the stem parts of BYZ No.1 and BYZ No.3 were lower than those in the apical parts, but they were still 8.8 times and 5.1 times those in WZS1.

Principal component analysis (PCA) roughly grouped all samples into three clusters, with PC1 and PC2 explaining 22.5% and 17.7% of the variance, respectively (Figure 1c). Consequently, the heat map failed to reveal notable differences in terms of cultivars or parts (Figure 1b), even though the content differences among the three grades of Wuzhi No. 2 were significantly evident. The limited separation observed in unsupervised PCA is not uncommon in flavoromics studies [19,20,21]. This indicated that the overall variation was distributed across multiple dimensions rather than concentrated in a few components.

However, the score plot still showed the general grouping trend: the two Baiyuzan cultivars were both concentrated along PC1, while the samples of Wuzhi No. 2 were mostly concentrated in the middle part of PC2. The apical and stem parts were located at the negative and positive ends of PC2, respectively. The biplot (Figure 1d) generated from PCA illustrated that α-thujene, hexathiane, naphthalene, and dimethyl trisulfide exhibited strong positive loadings on PC1, suggesting that they were major contributors to the variance captured by this component and were associated with Baiyuzan samples. Conversely, benzothiazole, 3,3-dimethylhexane, and hexanol had prominent negative loadings on PC1, indicating their significant roles in differentiating samples along the respective principal axes.

To verify the reliability of the HS-SPME-GC-MS results, identification and quantification of 2,4-di-tert-butylphenol, 1-octen-3-ol, hexadecane and benzothiazole was carried out by using authentic standards and external standard calibration curves (Figure 1e). Benzothiazole was not detected in any of the lotus sprouts. As for hexadecane, no significant difference in its content was observed among the three tested cultivars. As shown in Figure 1e, lotus sprouts contained a relatively high amount of 2,4-di-tert-butylphenol, with an average content of up to 403.9 μg/g. 2,4-di-tert-butylphenol has a distinct aromatic odor and is widely present in plants [22]. Existing studies have demonstrated that 2,4-di-tert-butylphenol not only contributes to the unique flavor of plant-derived foods but also exhibits certain biological activities, such as antioxidant and anti-inflammatory effects [23], which may be one of the key factors underlying the flavour characteristic of lotus sprouts, but the functional activities of the relevant substances in the lotus sprout still need to be further verified. Similarly, the average content of 1-octen-3-ol in lotus sprouts was 15.9 μg/g, with higher levels in the stems than in the apical parts. 1-Octen-3-ol, a volatile compound described as having fishy and mushroom-like notes, was significantly lower in the two BYZ cultivars than in Wuzhi No. 2 [24]. However, whether this difference translates into perceptible sensory differences requires further sensory evaluation.

### 3.3. Untargeted Metabolomics

#### 3.3.1. Comparison of the Apical and Stem Parts of Lotus Sprout

**Figure 2:**
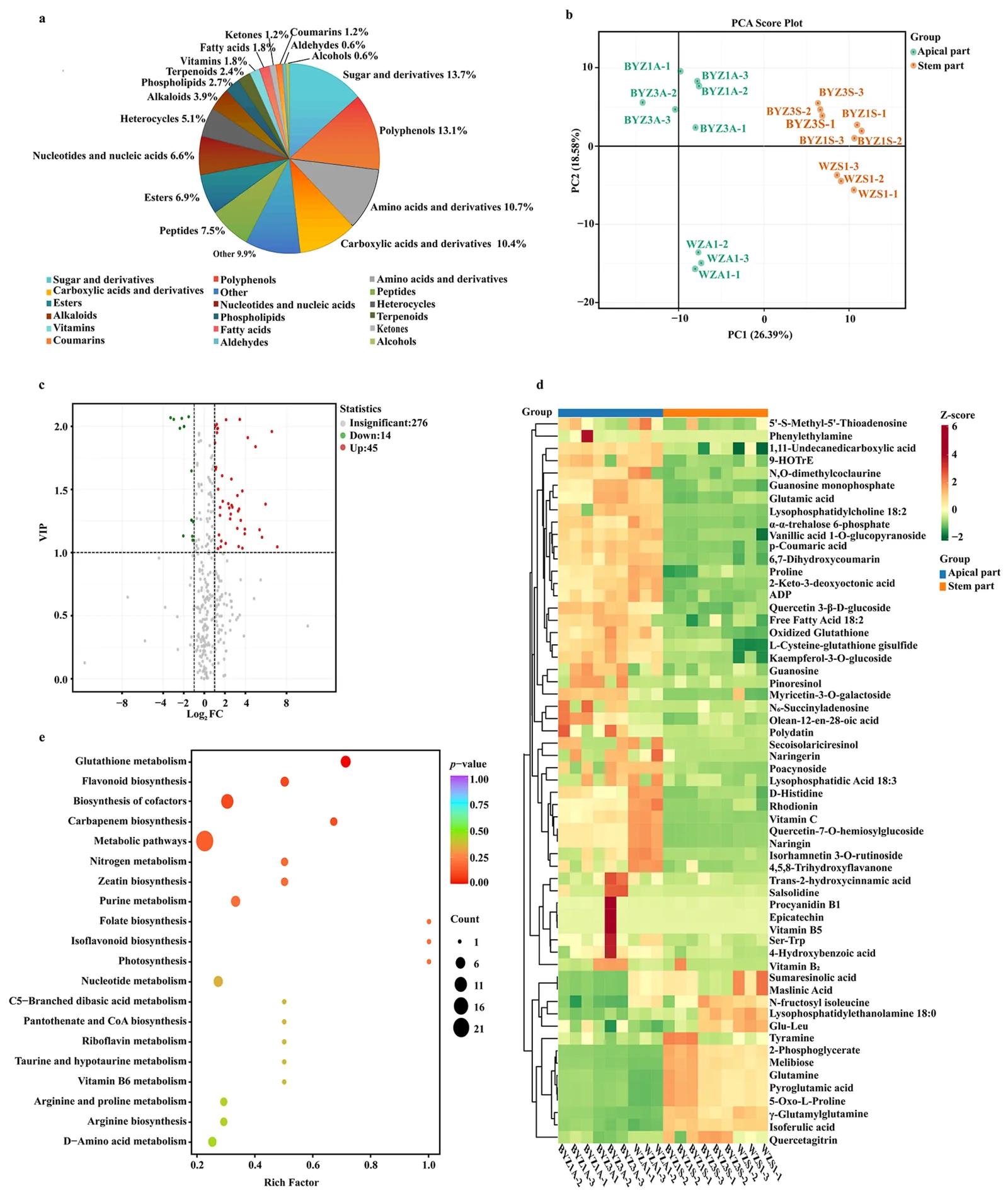
Metabolomics profiles of the apical and stem parts of lotus sprout. (**a**) Pie chart of all metabolites detected in lotus sprouts. (**b**) PCA score plot of the apical vs. stem parts. (**c**) Volcano plot of differentially accumulated metabolites (DAMs). (**d**) Heatmap analysis of all DAMs. (**e**) Top 20 KEGG pathways for DAMs in the apical vs. stem parts. WZA1 and WZS1 represent the apical and stem sections of Wuzhi No. 2 at Grade 1, respectively; WZA2 and WZS2 correspond to Grade 2, and WZA3 and WZS3 correspond to Grade 3. BYZ1A and BYZ1S are the apical and stem sections of Baiyuzan No. 1 at Grade 1, and BYZ3A and BYZ3S are those of Baiyuzan No. 3 at Grade 1.

Untargeted metabolomics analysis identified a total of 335 compounds, including 254 in positive ion mode and 81 in negative ion mode (Table S5). As shown in Figure 2a, these compounds were primarily categorized into 18 classes, mainly including sugars and derivatives (13.7%), polyphenols (13.1%), amino acids and derivatives (10.7%), carboxylic acids and derivatives (10.4%), and peptides (7.5%). PCA showed the overall difference among the three cultivars (Figure 2b). The total contribution rate of the three principal components was 54.5%, indicating that they could reflect most of the information of the samples (Figure 2b). The apical parts of BYZ No. 1 and BYZ No. 3 were closer to each other and were concentrated in the positive part of the second principal component, while they were far apart from Wuzhi No. 2, which was concentrated in the negative part. However, the stems of the three varieties were all concentrated in the positive part of the first principal component (Figure 2b), indicating that the metabolic composition of the stem sections of different cultivars shared higher similarity, while the apical sections showed more distinct cultivar-specific differences.

The differentially accumulated metabolites (DAMs) between the apical and stem parts were identified, with 45 metabolites accumulating more in the apical part and 14 accumulating more in the stem part (Figure 2c). A heatmap generated for the differential metabolites further visualized these changes and clearly indicated that most of these compounds were present in relatively high concentrations in the apical parts. These included certain amino acids, vitamins, and polyphenols, such as glutamic acid, proline, vitamin C, and vitamin B5, as well as 4-hydroxybenzoic acid, p-coumaric acid, salsolidine, and epicatechin (Figure 2d). KEGG pathway analysis demonstrated that the DAMs were significantly enriched in metabolic pathways (Figure 2e). Among them, glutathione metabolism, flavonoid biosynthesis, and biosynthesis of cofactors emerged as the top three pathways with significant enrichment.

#### 3.3.2. Comparison of the Different Cultivars of Lotus Sprout

**Figure 3:**
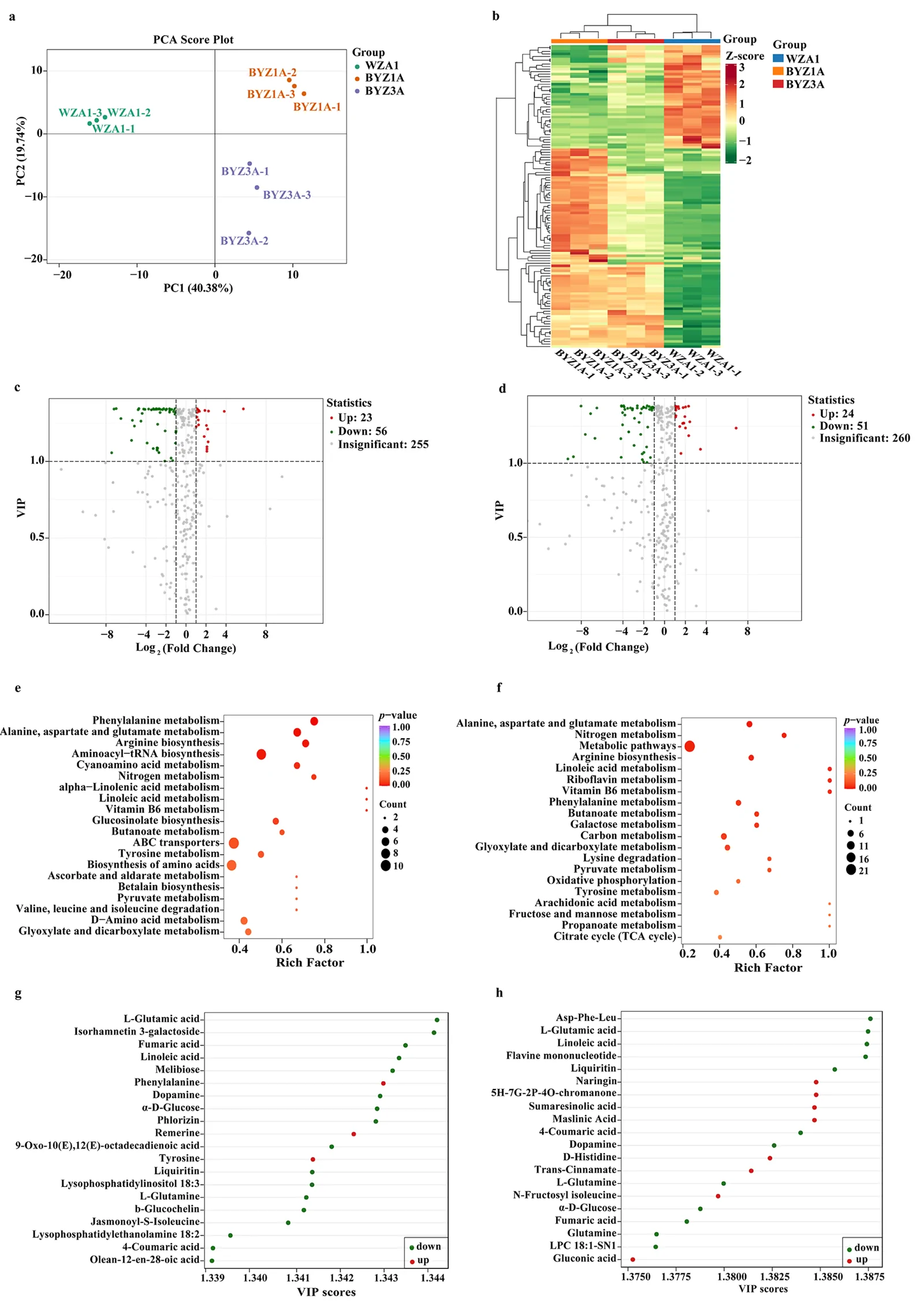
Metabolomics profiles of the apical parts of the three lotus sprout cultivars. PCA score plot (**a**) and heatmap analysis (**b**). Volcano plot of Wuzhi No. 2 vs. BYZ No. 1 (**c**) and Wuzhi No. 2 vs. BYZ No. 3 (**d**). Top 20 KEGG pathways of Wuzhi No. 2 vs. BYZ No. 1 (**e**) and Wuzhi No. 2 vs. BYZ No. 3 (**f**). Top 20 DAMs of Wuzhi No. 2 vs. BYZ No. 1 (**g**) and Wuzhi No. 2 vs. BYZ No. 3 (**h**). WZA1, BYZ1A, and BYZ3A: apical sections of Wuzhi No. 2, BYZ No. 1, and BYZ No. 3 at Grade 1, respectively.

In this study, the metabolites of the first-grade samples of the three most common cultivars of lotus sprout were compared. The PCA score plot indicated that the apical portions of Wuzhi No. 2, BYZ No. 1, and BYZ No. 3 were distributed in three quadrants (Figure 3a). Hierarchical clustering analysis and heatmap visualization revealed distinct metabolic profiles among the three cultivars, indicating that each cultivar possesses a unique metabolite composition contributing to its specific flavor and nutritional traits. Overall, the relative contents of most compounds were higher in BYZ No. 1 and BYZ No. 3 compared to those in Wuzhi No. 2 (Figure 3b). Orthogonal partial least squares-discriminant analysis (OPLS-DA) was applied to distinguish the differential accumulation metabolites (DAMs). All models demonstrated excellent predictive ability and no randomly permuted model could reproduce the separation performance of the original model, demonstrating the reliable group discrimination and absence of overfitting. The bar plots and 200-permutation test plots of all OPLS-DA models are listed in Figure S1.

For the comparison between Wuzhi No. 2 and BYZ No. 1, 23 DAMs were more abundant in the apical part of Wuzhi No. 2, and 56 DAMs were more abundant in BYZ No. 1 (Figure 3c). Similarly, in the comparison of Wuzhi No. 2 vs. BYZ No. 3, 24 DAMs accumulated to higher levels in the apical part of Wuzhi No. 2, and 51 DAMs accumulated to higher levels in BYZ No. 3 (Figure 3d). This suggests that the two BYZ cultivars possess a richer metabolic profile that differentiates them from Wuzhi No. 2.

KEGG pathway analysis demonstrated that these DAMs were mainly enriched in amino acid metabolism, including phenylalanine, alanine, aspartate, arginine, etc. (Figure 3e,f). These findings suggest that cultivar-specific quality differences in the apical part of lotus sprouts were mainly attributable to primary metabolism, especially nitrogen metabolism. Moreover, the apical part, as a meristematically active region, displayed distinct amino acid profiles, which may contribute to differences in nutritional value and flavor formation among cultivars.

Beyond amino acid metabolism, the top 20 DAMs included several organic acids and flavone glycosides that accumulated to higher levels in the two BYZ cultivars, including fumaric acid, 4-coumaric acid, linoleic acid and liquiritin (Figure 3g,h). Collectively, the BYZ cultivars exhibited a more active overall metabolism, which likely underlies their more intense flavor and greater nutritional density. This was also in agreement with the observed higher abundance of volatile compounds in the BYZ cultivars (Figure 1a).

**Figure 4:**
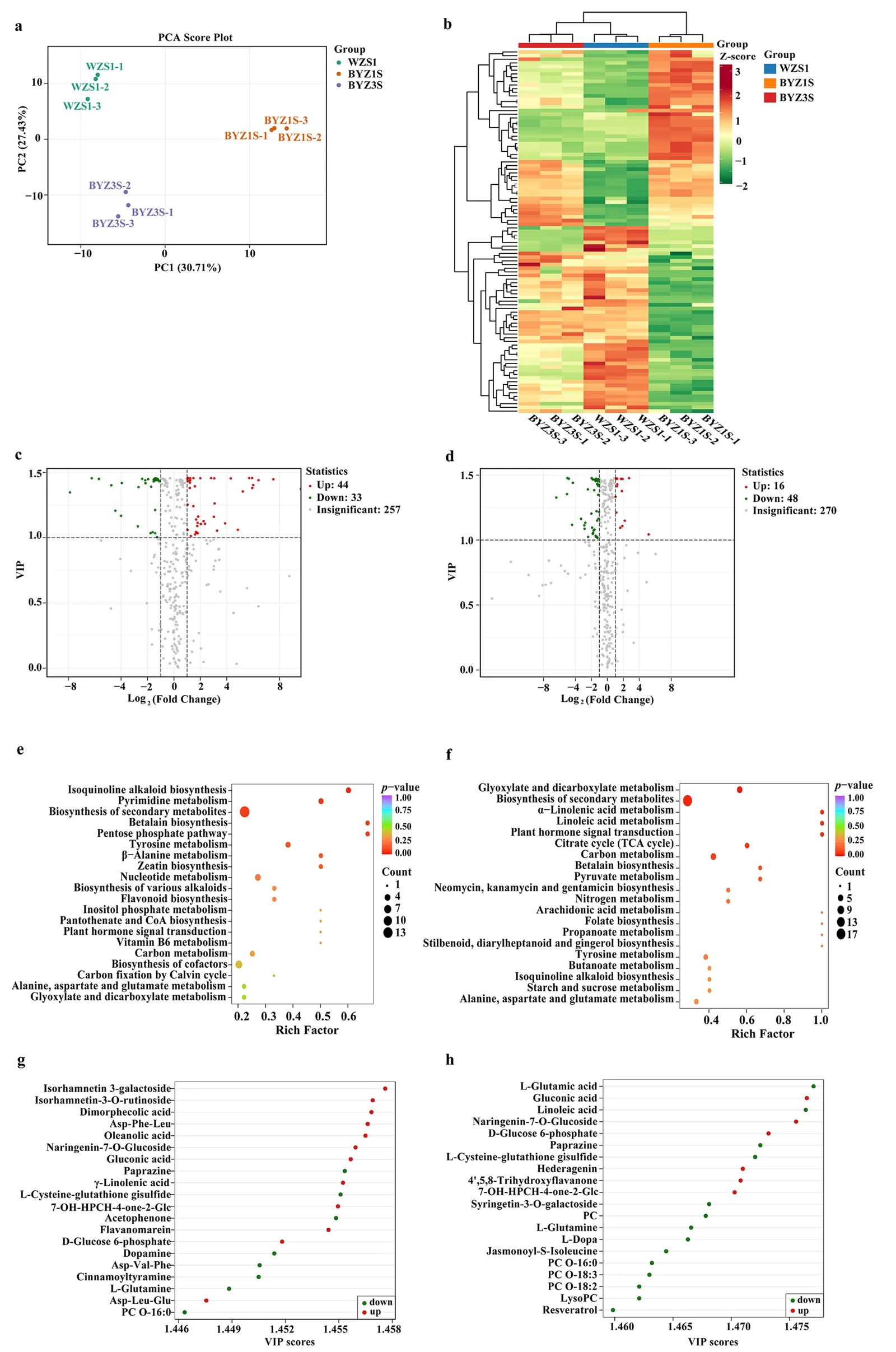
Metabolomics profiles of the stem parts of the three lotus sprout cultivars. PCA score plot (**a**) and heatmap analysis (**b**). Volcano plot of Wuzhi No. 2 vs. BYZ No. 1 (**c**) and Wuzhi No. 2 vs. BYZ No. 3 (**d**). Top 20 KEGG pathways of Wuzhi No. 2 vs. BYZ No. 1 (**e**) and Wuzhi No. 2 vs. BYZ No. 3 (**f**). Top 20 DAMs of Wuzhi No. 2 vs. BYZ No. 1 (**g**) and Wuzhi No. 2 vs. BYZ No. 3 (**h**). WZS1, BYZ1S, and BYZ3S: stem sections of Wuzhi No. 2, BYZ No. 1, and BYZ No. 3 at Grade 1, respectively.

The stem parts of Wuzhi No. 2, BYZ No. 1, and BYZ No. 3 were also clearly separated, accounting for 58.14% of the total variance (Figure 4a). Hierarchical clustering analysis and heatmap visualization indicated that the stem parts of Wuzhi No. 2 and BYZ No. 3 were grouped together, while the relative contents of the compounds were markedly different between Wuzhi No. 2 and BYZ No. 1 (Figure 4b). In detail, 44 DAMs accumulated more in the stem part of Wuzhi No. 2, and 33 DAMs accumulated more in BYZ No. 1 (Figure 4c). Only 16 DAMs accumulated more in Wuzhi No. 2, while 48 DAMs accumulated more in BYZ No. 3 (Figure 4d).

In contrast to the apical part, KEGG pathway analysis revealed that the DAMs in the stem part were mainly enriched in secondary metabolism, including alkaloids and flavonoids (Figure 4e,f). Representative DAMs in these pathways included nuciferine, betaine, salsolidine, isorhamnetin-3-galactoside, naringenin-7-O-glucoside and flavanomarein. These results indicate a distinct tissue-specific metabolic division in lotus sprouts: the apical part is primarily associated with growth vigor and nitrogen utilization, whereas the stem part is more involved in defense responses, flavor formation, and functional quality. Furthermore, analysis of the top 20 DAMs demonstrated that the metabolic differences in the stem part were also associated with oxidative stress responses, and carbon metabolism (Figure 4g,h).

#### 3.3.3. Comparison of the Different Grades of Lotus Sprout

**Figure 5:**
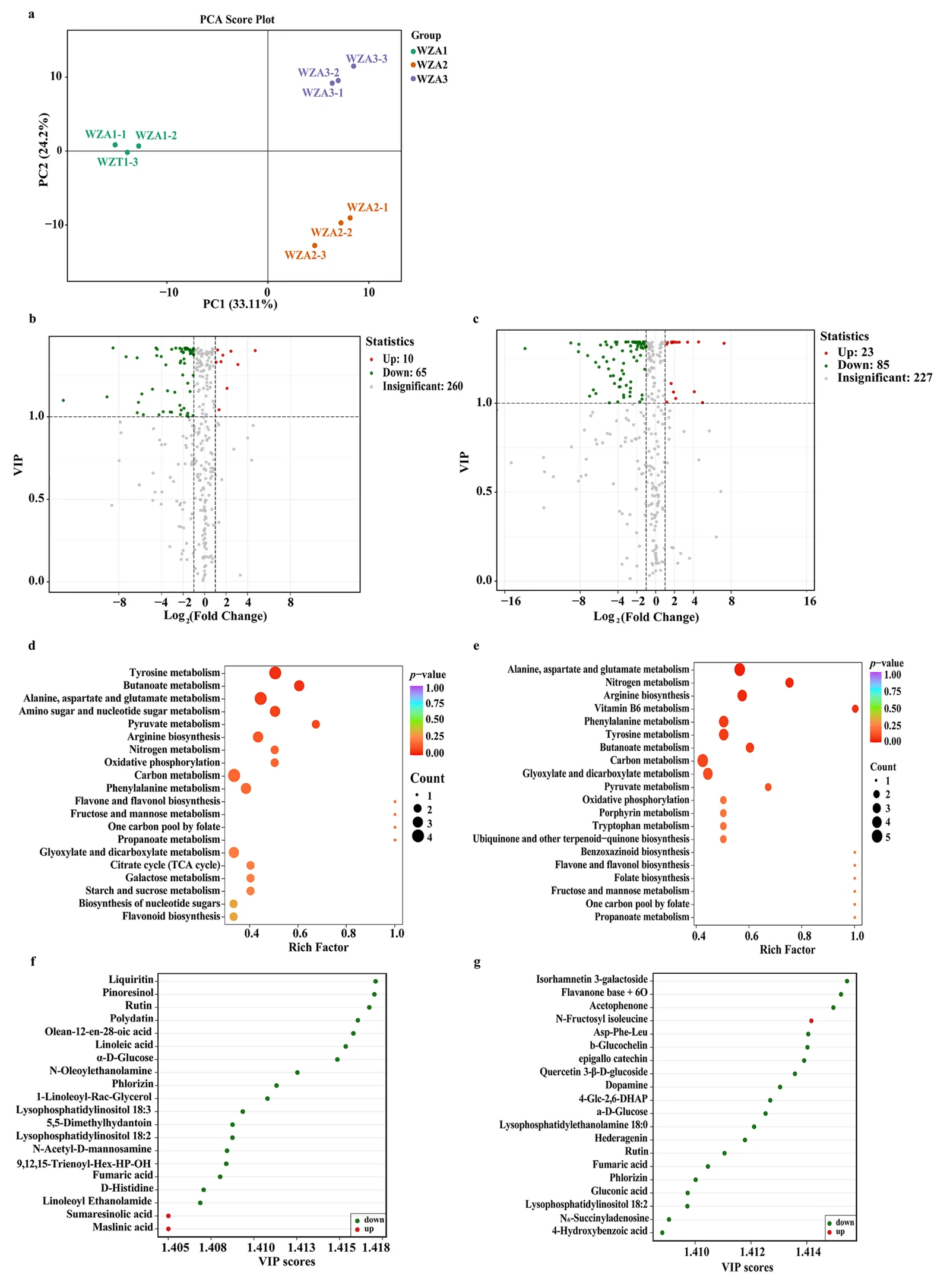
Metabolomics profiles of the apical parts of the three grades of Wuzhi No. 2. PCA score plot (**a**). Volcano plot of grade 1 vs. grade 2 (**b**) and grade 1 vs. grade 3 (**c**). Top 20 KEGG pathways of grade 1 vs. grade 2 (**d**) and grade 1 vs. grade 3 (**e**). Top 20 DAMs of grade 1 vs. grade 2 (**f**) and grade 1 vs. grade 3 (**g**). WZA1, WZA2, and WZA3: apical sections of Wuzhi No. 2 at grades 1, 2, and 3, respectively.

To further investigate metabolite differences among the three commercial grades of Wuzhi No. 2, PCA and OPLS-DA were performed. The PCA score plot showed a clear separation among the apical part of three grades, with grade 1 samples clustering primarily in the negative region of PC1 (Figure 5a). Moreover, most DAMs were present at relatively low concentrations in grade 1. Compared to grade 1, a total of 65 DAMs showed higher accumulation in grade 2 (Figure 5b), and 85 DAMs showed higher accumulation in grade 3 (Figure 5c), indicating a progressive increase in the number of up-regulated metabolites with increasing maturity.

KEGG pathway enrichment analysis revealed that the DAMs in the two comparison groups were mainly enriched in amino acid metabolism (Figure 5d,e). In the grade 1 vs. grade 2 comparison, a total of 9 DAMs were involved in 11 amino acid metabolism pathways, including L-glutamine, L-glutamic acid, fumaric acid, succinic acid, salidroside, dopamine, N-acetyl-L- phenylalanine, and 3-hydroxybenzoic acid. For grade 1 vs. grade 3, 12 DAMs were involved in 12 amino acid metabolism pathways. In addition to the above 9 compounds, they additionally contained tyramine, trans-cinnamate, indole, and 3-methylindole, but lacked salidroside. These results indicate that as the commercial grade declines from grade 1 to grade 3, the metabolic divergence becomes progressively more complex, particularly affecting aromatic amino acid metabolism and downstream pathways related to flavor and defense.

Analysis of the top 20 DAMs between grade 1 and grade 2 revealed that secondary and lipid metabolism were more active in grade 2 samples (Figure 5f). Metabolites with antioxidant, anti-inflammatory, and signaling functions, such as liquiritin, pinoresinol, rutin, and polydatin, accumulated to higher levels in grade 2. Similarly, in the grade 1 vs. grade 3 comparison, most of the top 20 DAMs showed higher abundance in grade 3, with the exception of N-fructosyl isoleucine (Figure 5g). N-fructosyl isoleucine is an Amadori compound, typically associated with freshness and sweetness. The higher levels of N-fructosyl isoleucine in grade 1 lotus sprouts align with their superior freshness and higher commercial value.

**Figure 6:**
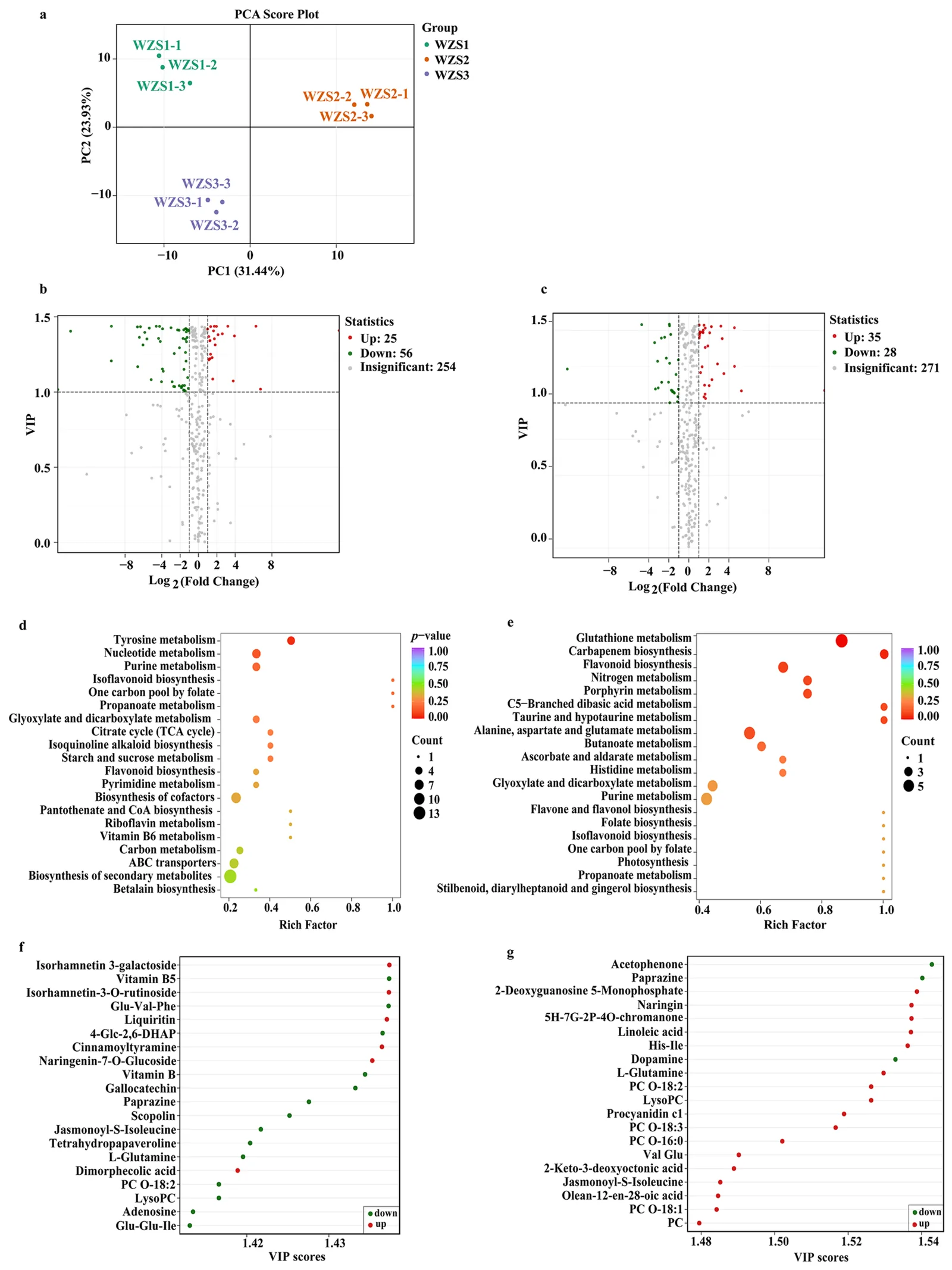
Metabolomics profiles of the stem parts of the three grades of Wuzhi No.2. PCA score plot (**a**). Volcano plot (**b**), top 20 KEGG pathways (**c**) and top 20 DAMs (**d**) in grade 1 vs. grade 2. Volcano plot (**e**), top 20 KEGG pathways (**f**) and top 20 DAMs (**g**) in grade 1 vs. grade 3. WZS1, WZS2, and WZS3: stem sections of Wuzhi No. 2 at grades 1, 2, and 3, respectively.

Metabolite differences among the stem parts of three commercial grades of Wuzhi No. 2 were further analyzed. PCA revealed a clear separation among the three grade groups, with the first two principal components accounting for 55.37% of the total variance (Figure 6a). No consistent accumulation pattern was observed for DAMs in the stem parts across grades. Specifically, when comparing grade 1 and grade 2, 25 DAMs showed higher accumulation in grade 1, whereas 56 DAMs showed higher accumulation in grade 2 (Figure 6b). In the comparison between grade 1 and grade 3, 35 DAMs were more abundant in grade 1, while 28 DAMs were more abundant in grade 3 (Figure 6c). This indicated that the metabolic changes in the stem part do not follow the same progressive accumulation trend as those in the apical part.

KEGG pathway enrichment analysis was performed between three grade of stem part, flavonoid biosynthesis was consistently enriched across both comparisons. Meanwhile, grade 1 vs. grade 2 additionally involved TCA cycle, starch/sucrose metabolism, and alkaloid pathways, while grade 1 vs. grade 3 uniquely enriched glutathione metabolism, ascorbate metabolism, nitrogen metabolism, and stilbenoid biosynthesis (Figure 6d,e). These results indicate that as the grade difference widens, the metabolic in the stem part transitions from alterations in energy and storage metabolism toward antioxidant and defense pathways.

Comparison of the top 20 DAMs between grade 1 and grade 2 revealed that 14 metabolites showed higher accumulation in grade 2, including B vitamins, peptides, and alkaloids. This indicated that the grade 2 samples were in a more mature physiological state, with more active pathways related to defense responses, energy metabolism, and signal transduction. In contrast, cinnamoyltyramine and dimorphecolic acid had higher content in grade 1. They were related to the cross-linking of the cell wall and the formation of the cuticle, which helped to maintain a tender texture.

Meanwhile, in the comparison of grade 1 and grade 3, 17 metabolites showed higher accumulation in grade 1. Among them, all DAMs showed fold changes greater than 2.0, with Procyanidin C1 reaching 23.6, suggesting a general decline in metabolic activity and cellular integrity in grade 3. This aligns with the fact that lower-grade lotus sprouts exhibit higher susceptibility to quality deterioration, including oxidative browning and loss of texture firmness. Moreover, three defense-related metabolites, acetophenone, paprazine, and dopamine, showed higher accumulation in grade 3, further clarifying that grade 3 samples had enhanced the defense against the aging process.

### 3.4. Comparison of the Kinds and Concentrations of Alkaloids

**Figure 7:**
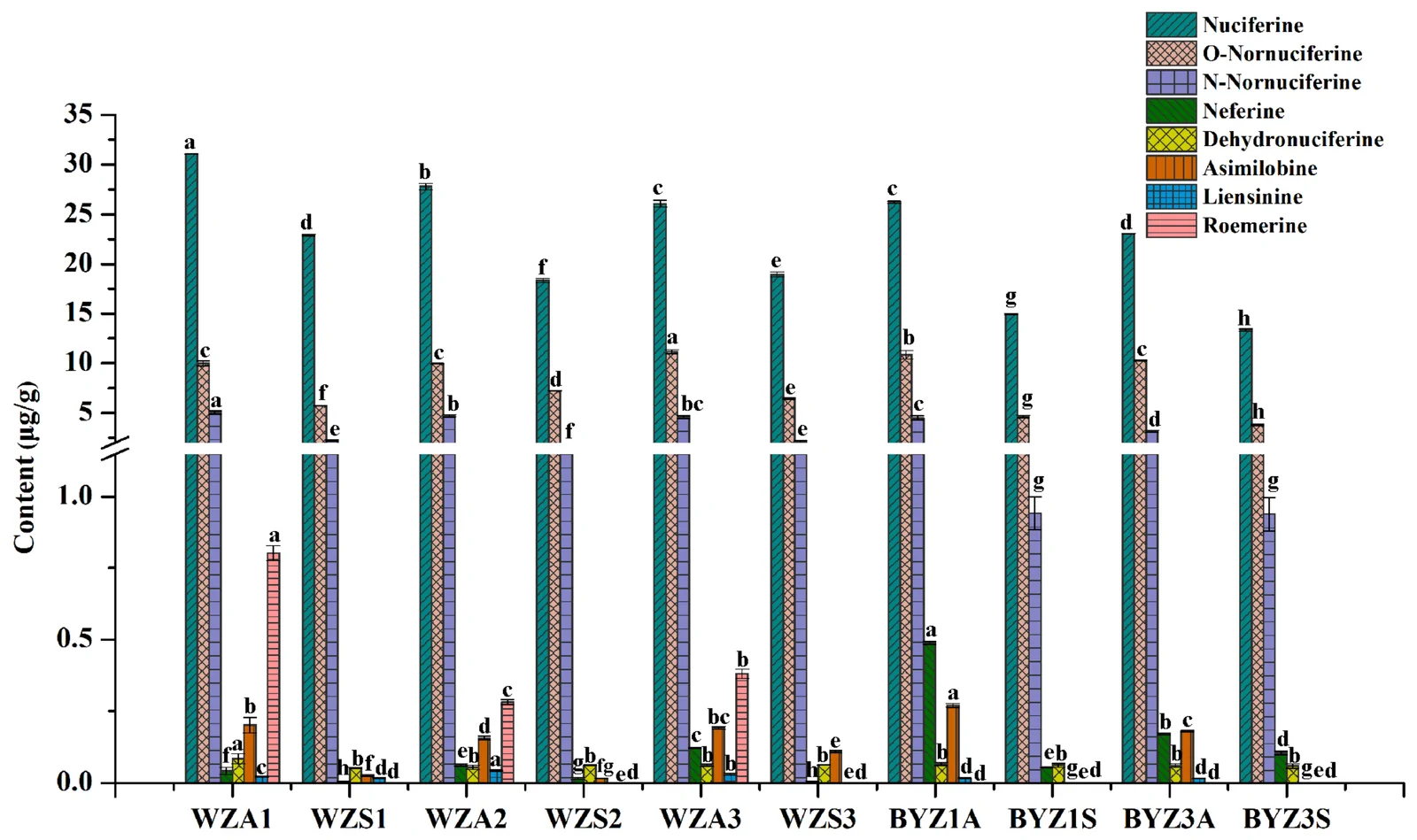
Amount of alkaloids detected in lotus sprouts by UPLC-MS/MS. Data are presented as mean ± standard deviation (SD) (*n* = 3), different lowercase letters indicate significant differences between groups (*p* < 0.05). WZA1 and WZS1 represent the apical and stem sections of Wuzhi No. 2 at Grade 1, respectively; WZA2 and WZS2 correspond to Grade 2, and WZA3 and WZS3 correspond to Grade 3. BYZ1A and BYZ1S are the apical and stem sections of Baiyuzan No. 1 at Grade 1, and BYZ3A and BYZ3S are those of Baiyuzan No. 3 at Grade 1.

To verify the metabolomics results, the kinds and concentrations of alkaloids in lotus sprouts from different cultivars and parts were quantified and compared using external standard calibration curves (Figure 7). The results indicated that a total of 8 alkaloids were identified in the lotus sprouts, including nuciferine and N-nornuciferine, identified through metabolomics analysis. Among these, nuciferine was the most abundant, accounting for over 60% of the total alkaloid content. Evidently, the contents of most alkaloids were significantly higher in the apical sections than in the stem sections, such as nuciferine, O-nornuciferine, neferine, and roemerine. Conversely, the differences in the contents of other alkaloids between the apical and stem sections were not statistically significant. Moreover, liensinine and roemerine were present at relatively low levels and mainly accumulated in the apical sections. Except for a small amount of liensinine detected in the WZS1 stem section, neither liensinine nor roemerine was detected in the stem sections of the other sample groups.

Notable differences were also observed in the types and contents of alkaloids among the three cultivars. Specifically, the BYZ1 cultivar exhibited excellent accumulation of neferine and O-nornuciferine. The Wuzhi No. 2 cultivar demonstrated a distinct advantage in roemerine accumulation, while the BYZ3 cultivar showed an intermediate profile between the two.

## 4. Discussion

In this study, the basic nutritional components, volatile flavor compounds, and metabolites of lotus sprouts across various cultivars and grades were first comprehensively compared. The nutritional components reveal a compartmentalization of nutrients, with the apical part being a concentrated source of proteins and phenolics, while the stem primarily serves as a sugar storage organ. Some research has shown that mature lotus rhizome exhibits stronger antioxidant capacity due to its phenolic composition [25]. During processing, the apical parts of lotus sprouts are often regarded as waste, yet they contain the highest total phenolic content, reaching up to 8.19 mg/g. This indicates that the apical parts of lotus sprouts have significant antioxidant potential.

Volatile flavor compounds (VOCs) are crucial for the aroma quality of vegetables and fruits. Numerous studies have been conducted on the volatile substances in lotus rhizomes [26,27]. Here, we present 46 compounds that initially describe the volatile flavor profile of lotus sprouts. With the exception of 1-hexanol and hexanal, α-thujene and sylvestrene, which have the distinctive scents of herbs and pine wood, may contribute to the unique aroma quality of the lotus sprout [28,29]. From the account of VOCs, the accumulation of flavor substances in tender lotus sprouts is less than that in mature lotus rhizomes. For example, a total of 99 volatile compounds have been detected in whole powders of lotus rhizomes (Elian No. 5), including 3-heptanol, citronellal, nonanoic acid, furfuryl propionate, and α-phellandrene [30], while 66 VOCs were detected in the boiled lotus rhizome from 14 cultivars [4]. On the other hand, the composition of VOCs in different varieties may also vary. For example, in the lotus sprouts of “Elian No. 5”, only 12 VOCs were detected [31]. Moreover, conducting headspace–gas chromatography–ion mobility spectrometry and electronic nose analysis will help to more intuitively reflect the differences in volatile compounds among different lotus rhizome samples [27]. However, although the lotus sprout is the tender underground rhizome formed during the early growth stage of lotus, research on its flavor substances is relatively limited. The qualitative and quantitative verification of more characteristic flavor compounds could help to not only explain the differences in flavor and quality between different lotus sprout cultivars, but also to provide potential marker compounds for subsequent cultivar improvement and quality evaluation of lotus sprouts.

The untargeted metabolomics analysis reveals a multifaceted metabolic landscape in lotus sprouts. The apical portion consistently emerges as a primary accumulation site for nutritionally and functionally significant metabolites, including amino acids, vitamins, and polyphenols. This spatial partitioning aligns with the apical bud’s role as an active growth center of plant, demanding enriched pools of precursors for biosynthesis and antioxidants for stress mitigation [32,33]. This is further evidenced by the enrichment of DAMs in glutathione metabolism and flavonoid biosynthesis. Further analysis of the time course of precursors and auxin may help to analyze the patterns of the growth and expansion of lotus rhizomes [29,34].

Cultivar-specific metabolic signatures were pronounced, with BYZ No. 1 and No. 3 generally exhibiting higher metabolite abundances than Wuzhi No. 2, particularly in organic acids, sugars, and amino acid pathways, which likely underpin their distinct flavor and nutritional profiles. In terms of the time of variety selection, BYZ No. 1 and No. 3 were selected 30 years later than Wuzhi No. 2. They are specialized varieties for lotus sprout selected in recent years to increase the yield [35,36]. Furthermore, there is metabolic divergence among commercial grades of Wuzhi No. 2. However, the apical part of samples with higher product grades tend to have lower metabolite contents. This is related to the shorter growth cycle, but it provides a direction for improving product quality through breeding and cultivation techniques.

As a traditional herb, lotus has been used in treating various diseases due to its abundant active compounds. Among these, alkaloids are considered the most significant pharmacological ingredients [37]. Current research has demonstrated that the alkaloids are mainly found in lotus flowers, leaves, and seeds [1,38]. Usually, the lotus rhizome has the lowest levels of alkaloids; only the knots of the lotus rhizome have a total alkaloid content of 1.33–14.48 mg/g [39].

In this study, 8 alkaloids in lotus sprouts were quantified using external standard calibration curves. Nuciferine was the most abundant component, reaching 30 μg/g (freeze-drying weight). Overall, the alkaloid content of the two BYZ cultivars was relatively low, which may contribute to a better taste [40]. However, this preference for high-quality lotus sprouts in the market may sacrifice some functional metabolites, including active alkaloids that have healthcare value. Therefore, optimizing the utilization of different cultivars and grades of lotus sprouts is particularly important for enhancing the overall development value of lotus sprout resources. In addition to alkaloids, untargeted metabolomics also revealed that lotus sprouts contain terpenoids and coumarin-type compounds. Future studies could establish a fully validated quantitative method to further support the utilization of active compounds in lotus sprouts.

## 5. Conclusions

In summary, this study provides a comprehensive metabolic characterization of lotus sprouts and reveals clear part-specific and cultivar-specific variations in their chemical composition. The apical portions exhibited higher levels of protein, total phenolics, and GABA than the stems, supporting the higher edible and functional value of this part. Volatile profiling identified 46 compounds, but the differences among groups were relatively small. Untargeted metabolomics identified 355 compounds, and further analysis showed that the differentially accumulated metabolites among parts and cultivars were significantly enriched in glutathione metabolism, flavonoid biosynthesis, and amino acid metabolism, highlighting these pathways as key contributors to quality variation. The two Baiyuzan cultivars generally accumulated higher levels of beneficial metabolites than Wuzhi No. 2, whereas higher-grade Wuzhi No. 2 samples tended to contain lower levels of certain active compounds. Further studies could explore optimizing extraction methods to promote the development of new functional products. In addition, eight alkaloids were quantified using external standard calibration curves, with nuciferine identified as the most abundant alkaloid (13.3–31.1 μg/g), providing a practical basis for quality evaluation. Collectively, these findings offer a metabolic framework for understanding lotus sprout quality and support the development of improved cultivars with better nutritional and sensory properties.

## Figures and Tables

**Table 1:** The appearance and grade information of lotus sprout samples.

No.	Appearance	Sample Name	Description
1	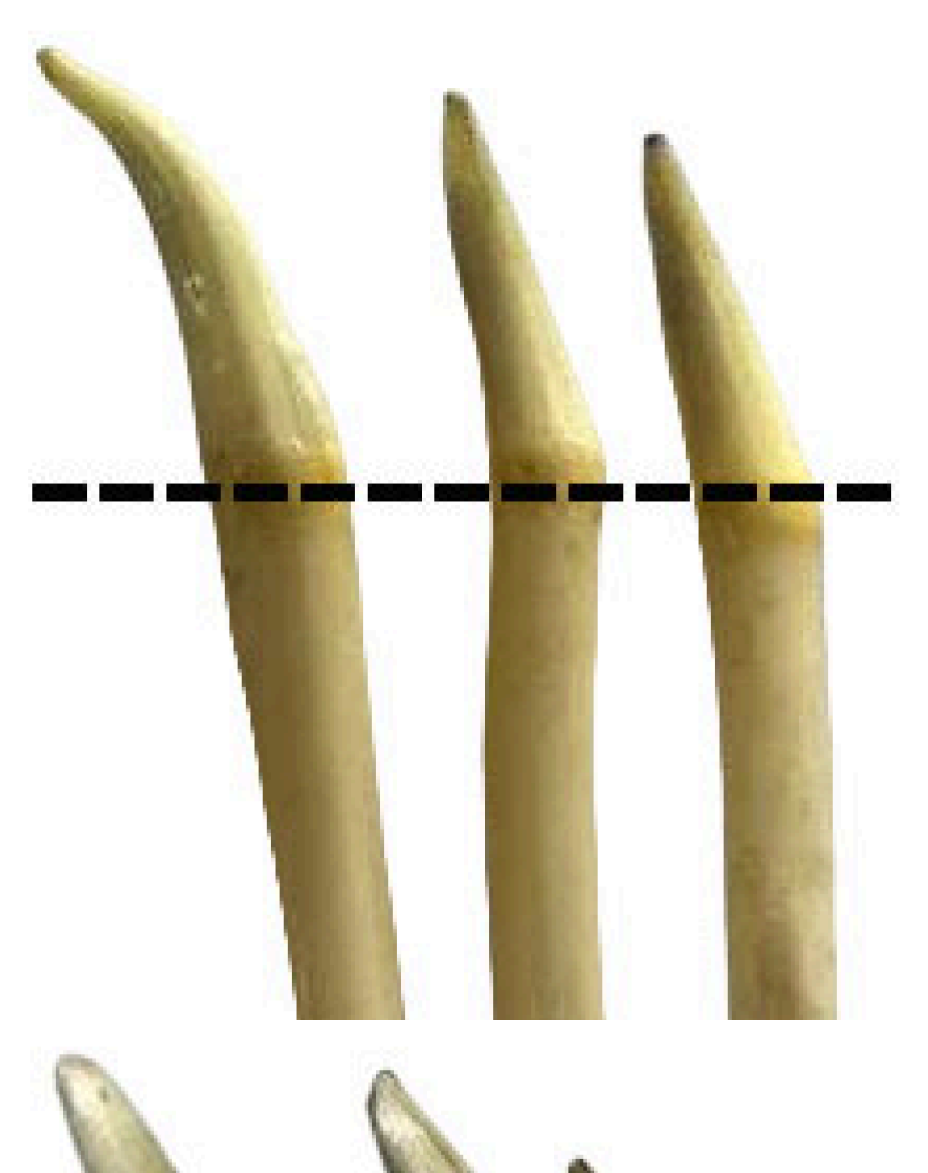	WZA1	The apical section of Wuzhi No. 2 at grade 1
2		WZS1	The stem section of Wuzhi No. 2 at grade 1
3	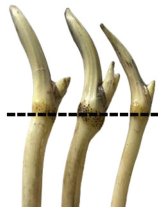	WZA2	The apical section of Wuzhi No. 2 at grade 2
4		WZS2	The stem section of Wuzhi No. 2 at grade 2
5	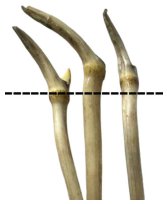	WZA3	The apical section of Wuzhi No. 2 at grade 3
6		WZS3	The stem section of Wuzhi No. 2 at grade 3
7	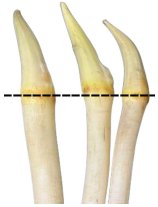	BYZ1A	The apical section of Baiyuzan No. 1 at grade 1
8		BYZ1S	The stem section of Baiyuzan No. 1 at grade 1
9	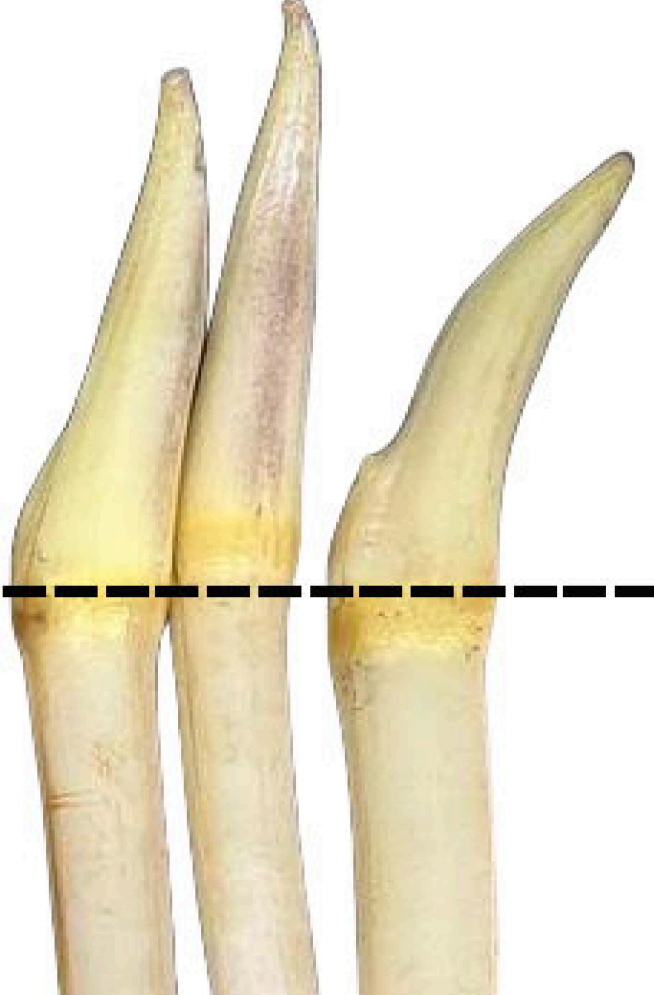	BYZ3A	The apical section of Baiyuzan No. 1 at grade 3
10		BYZ3S	The stem section of Baiyuzan No. 1 at grade 3

Note: The dotted lines represent the dividing lines between different parts of the lotus sprout samples. The upper part is the apical section of the lotus sprout, and the lower part is the stem section.

**Table 2:** Analysis of the content of basic nutritional components.

Samples	Moisture (%)	Protein (%)	FAA (%)	GABA (%)	Reducing Sugar (%)	Soluble Sugar (%)	TPC-W (mg/g)	TPC-M (mg/g)
WZA1	94.3 ± 1.0 ^ab^	24.73 ± 0.02 ^b^	0.84 ± 0.00 ^a^	0.20 ± 0.00 ^bc^	0.102 ± 0.004 ^e^	0.114 ± 0.004 ^f^	6.54 ± 0.14 ^a^	7.29 ± 0.13 ^b^
WZS1	95.9 ± 0.7 ^a^	18.31 ± 0.09 ^e^	0.82 ± 0.23 ^ab^	0.27 ± 0.08 ^a^	0.274 ± 0.006 ^c^	0.258 ± 0.004 ^d^	4.76 ± 0.12 ^d^	6.36 ± 0.14 ^c^
WZA2	93.8 ± 0.8 ^b^	23.05 ± 0.14 ^d^	0.58 ± 0.10 ^cd^	0.15 ± 0.03 ^cd^	0.080 ± 0.003 ^f^	0.096 ± 0.004 ^g^	5.67 ± 0.44 ^c^	6.61 ± 0.12 ^c^
WZS2	95.1 ± 0.6 ^ab^	15.60 ± 0.01 ^f^	0.30 ± 0.10 ^e^	0.07 ± 0.02 ^e^	0.343 ± 0.014 ^b^	0.328 ± 0.002 ^b^	4.13 ± 0.17 ^d^	5.89 ± 0.18 ^d^
WZA3	94.6 ± 1.1 ^ab^	27.47 ± 0.18 ^a^	0.84 ± 0.09 ^a^	0.24 ± 0.03 ^ab^	0.074 ± 0.003 ^g^	0.081 ± 0.004 ^h^	4.90 ± 0.84 ^d^	8.13 ± 0.30 ^a^
WZS3	94.9 ± 1.2 ^ab^	14.64 ± 0.02 ^g^	0.61 ± 0.18 ^bcd^	0.16 ± 0.04 ^cd^	0.339 ± 0.003 ^b^	0.341 ± 0.004 ^a^	4.14 ± 0.09 ^d^	6.76 ± 0.33 ^c^
BYZ1A	93.3 ± 0.6 ^b^	23.42 ± 0.41 ^c^	0.36 ± 0.08 ^e^	0.08 ± 0.02 ^e^	0.143 ± 0.003 ^d^	0.168 ± 0.003 ^e^	8.09 ± 0.18 ^a^	8.19 ± 0.25 ^a^
BYZ1S	94.7 ± 0.8 ^ab^	15.69 ± 0.27 ^f^	0.43 ± 0.04 ^de^	0.08 ± 0.00 ^e^	0.374 ± 0.013 ^a^	0.346 ± 0.008 ^a^	4.85 ± 0.91 ^d^	7.27 ± 0.08 ^b^
BYZ3A	93.4 ± 0.7 ^b^	23.07 ± 0.32 ^d^	0.71 ± 0.13 ^abc^	0.14 ± 0.03 ^d^	0.140 ± 0.005 ^d^	0.166 ± 0.006 ^e^	7.72 ± 0.46 ^a^	7.54 ± 0.23 ^b^
BYZ3S	94.7 ± 2.0 ^ab^	16.28 ± 0.09 ^e^	0.37 ± 0.07 ^e^	0.11 ± 0.02 ^de^	0.326 ± 0.011 ^b^	0.304 ± 0.021 ^c^	4.33 ± 0.08 ^d^	7.28 ± 0.55 ^b^

Note: WZA1 and WZS1 represent the apical and stem sections of Wuzhi No. 2 at Grade 1, respectively; WZA2 and WZS2 correspond to Grade 2, and WZA3 and WZS3 correspond to Grade 3. BYZ1A and BYZ1S are the apical and stem sections of Baiyuzan No. 1 at Grade 1, and BYZ3A and BYZ3S are those of Baiyuzan No. 3 at Grade 1. Data are presented as mean ± standard deviation (SD) (*n* = 3), different lowercase letters indicate significant differences between groups (*p* < 0.05). FAA: free amino acid, TPC-W: total phenolic content extracted with water, TPC-M: total phenolic content extracted with methanol.

## Data Availability

The data presented in this study are available upon request to the corresponding author.

## Supplementary Materials

The following supporting information can be downloaded at: https://www.mdpi.com/article/10.3390/foods15213965/s1, Table S1: Reference calibration curves and ion parameters; Table S2: Mass spectrometry MRM conditions; Table S3: Standards and reference calibration curves for alkaloid analysis; Table S4: The 46 volatile compounds identified in lotus sprout samples; Table S5: The 355 compounds identified by untargeted metabolomics in lotus sprout samples; Figure S1: The OPLS-DA model parameter bar plots and 200-permutation test plots.

## References

[1] Huang J., He G., Wu L., Ma P., Xu L., Sun L., Xiao P. (2025). The edible lotus (*Nelumbo nucifera* Gaertn.) and its byproducts as valuable source of natural antioxidants: A review of phytochemicals, health benefits, safety and food applications. Future Foods.

[2] Liu L., Han J., Tian H., Liu Z., Wang X., Wang P., Li X., Gao W. (2025). A review on the progress of research on the chemical composition and pharmacological effects of lotus leaf. Food Chem..

[3] Ma S., Lei Q., Jiang X., Mao S., Huang Z., Zhou T., Zheng B., Zhou F., Lu X. (2025). The aroma evolution in lotus (*Nelumbo nucifera* Gaertn.) seed juice: A comprehensive analysis of processing and low-temperature storage effects. Food Chem..

[4] Duan R., Chen X., Chen X., Li J., Yan S. (2025). Sensory and flavor profiling of lotus rhizome pork rib soup: Identification of optimal cultivars using HS-SPME-GC/MS, E-nose, and E-tongue analyses. Food Chem..

[5] Cao Y., Yan S., Li J., Wang Q. (2021). Major factors affecting non-enzymatic browning of boiled lotus sprout during storage. Food Sci..

[6] Wang C., Wang S., Chang T., Shi L., Yang H., Shao Y., Feng W., Cui M. (2013). Efficacy of lactic acid in reducing foodborne pathogens in minimally processed lotus sprouts. Food Control.

[7] Zhao D., Mei D., Zeng X., Zeng L. (2019). Breeding of a new Oudai cultivar Huagui No.1 and its key cultivation techniques. J. Chang. Veg..

[8] Xu C., Chen X., Duan R., Liu Y., Yan S., Li J. (2026). Effects of different storage conditions on postharvest characteristics and microbial community of lotus sprouts. Food Chem..

[9] Yang S., Du J., Zhu Q., Chen M., Guo J., Wu Y., Cui L. (2023). Optimization of color protection formula for boiled lotus root strips. Mod. Agric. Sci. Technol..

[10] Younis I.Y., Farag M.A., Elgamal A.M., Mohsen E. (2023). Untargeted metabolites profiling of volatile and non-volatile components of Egyptian lotus (*Nelumbo nucifera* Gaertn.) using UHPLC/PDA/ESI-MS and solid-phase microextraction (SPME) GC/MS in relation to its antiaging and anti-inflammatory effects. Ind. Crops Prod..

[11] Ren H., Li Z., Zhou Q., Zhao H., Ma D., Guo X., Cai Z., Li Y., Zhang Z., Zheng Y. (2025). Multi-omics analysis of microbial spatiotemporal succession and metabolite differences in pit mud of varying cellar ages and spatial positions. Food Chem. X.

[12] Ren H., Peng D., Liu M., Wang Y., Li Z., Zhao H., Zheng Y., Liu Y., Feng X. (2025). Dynamic changes in chemical components, volatile profile and antioxidant properties of *Xanthoceras sorbifolium* leaf tea during manufacturing process. Food Chem..

[13] Liu J., Peng J., Yang J., Wang J., Peng X., Yan W., Zhao L., Peng L., Zhou Y. (2023). Comparative analysis of the physicochemical properties and metabolites of farinose and crisp lotus roots (*Nelumbo nucifera* Gaertn.) with different geographical origins. Foods.

[14] Mo C., Zhu Y. (2015). Absorptive Properties of Lotus Sprout for Fat and Cholesterol. J. Anhui Agric. Sci..

[15] Zhou M., Zheng X., Zhu H., Li L., Zhang L., Liu M., Liu Z., Peng M., Wang C., Li Q. (2021). Effect of *Lactobacillus plantarum* enriched with organic/inorganic selenium on the quality and microbial communities of fermented pickles. Food Chem..

[16] Luo Y., Ouyang L., Zhang S., Wang Y., Yao P., Liu Y., Chen X., Yuan L., Wu Z. (2025). Insights into VOCs variation derived from Chinese black truffles (*Tuber indicum*) between grades via E-nose, HS-SPME-GC-MS, and HS-GC-IMS. Food Chem. X.

[17] Duan W., Yao Z., Qiao C., Meng J., Sun S., Pan L., Niu L., Cui G., Wang Z., Zeng W. (2026). Combined volatile metabolome and transcriptome analysis of 60 peach cultivars provide new insights into the formation of aroma and the identification of associated genes. Hortic. Plant J..

[18] Sarkar R., Kumar S., Singh R. (2026). UHPLC-PDA-MS based simultaneous characterization of five isoquinoline alkaloids and mineral profiling in stem of four Tinospora species from India. Microchem. J..

[19] Hao C., Yu Y., Zhang X., Dong G., Liu Y., Chen S. (2022). Nontarget metabolites of rhizomes of edible sacred lotus provide new insights into rhizome browning. J. Food Qual..

[20] Li X., Li G., Wang H., Liu C., Rong C., Song F., Chen C., Wu J. (2025). Characterization of volatile flavor profiles in three peach cultivars during postharvest storage at various temperatures using HS-SPME-GC–MS. Food Chem. X.

[21] Lu P., Wang C., Zhang Y., Jin H., Wu S., Ding X., Zhang H. (2025). Diversity of volatile profiles and nutritional traits among 29 cucumber cultivars. Foods.

[22] Wang F., Feng H., Zheng Y., Liu R., Dong J., Wu Y., Chen S., Zhang B., Wang P., Yan J. (2025). Aroma analysis and biomarker screening of 27 tea cultivars based on four leaf color types. Food Res. Int..

[23] Zhao F., Wang P., Lucardi R.D., Su Z., Li S. (2020). Natural Sources and Bioactivities of 2,4-Di-Tert-Butylphenol and Its Analogs. Toxins.

[24] Liu Y., Wang Y., Liu Z., Li C., Yu Q., Xie J., Long Y., E Q., Chen Y. (2026). Flavor modulation of braised He Bao carp (*Cyprinus carpio*) by natural plant spices: Insights from GC–MS, GC–IMS, E-nose, E-tongue, and molecular docking analyses. Food Biosci..

[25] Zhao R., Peng K., Yi Y., Sun Y., Jiang X., Wang H. (2024). Heating alters the nutritional and antioxidant characteristics of lotus root. LWT.

[26] Shi Q., Wang Y., Han C., Guo X., Fu M., Tian S., Xin X. (2025). Effect of epsilon-poly-L-lysine treatment on swelling, microbial growth and physicochemical quality of vacuum-packed lotus root. Food Control.

[27] Li Y., Peng K., Wang H., Sun Y., Jiang X., Yi Y. (2024). Physicochemical properties and volatile compounds of whole lotus root powders prepared by different drying methods. LWT.

[28] Desai S., Singh Sharanagat V., Kumar Nema P., Mani S., Alam Khan Z. (2024). Assessment of microwave roasting-induced changes in composition and signature flavor compounds of black pepper (*Pepper nigrum* L.). Microchem. J..

[29] Feng J., Yu F., Lu P., Yang Y., Li L., Yang L., Kang C., Yang L. (2025). Morphological observation and transcriptome profiling of axillary buds with different identities in response to gibberellin in woodland strawberry. Plant Physiol. Biochem..

[30] Jia C., Li Y., Sun Y., Jiang X., Wang H., Peng K., Yi Y. (2025). Flavor variation during the processing of lotus root whole powder: A combination of hot air drying, lysine supplementation and baking. Food Chem. X.

[31] Zhang X., Ding R., Wu Y., Wu Z., Chen M., Liu Y., Guo X., Gong Z. (2025). Comprehensive evaluation of nutritional quality and flavor properties of Caidian characteristic lotus sprout. Food Mach..

[32] Xue Z., Liu L., Zhang C. (2020). Regulation of shoot apical meristem and axillary meristem development in plants. Int. J. Mol. Sci..

[33] Liu M., Yang L., Guo J., Wang H., Yu S., Wang P., Cui X., Yang Y., Liu Y. (2025). Single-cell transcriptome atlas of Panax notoginseng embryonic shoot apex: Insights into developmental regulation and triterpene saponin biosynthesis. Ind. Crops Prod..

[34] Sorce C., Lombardi L., Giorgetti L., Parisi B., Ranalli P., Lorenzi R. (2009). Indoleacetic acid concentration and metabolism changes during bud development in tubers of two potato (*Solanum tuberosum*) cultivars. J. Plant Physiol..

[35] Lu B., Ji C., Heng D., Fang J. (1999). New variety of lotus root—“Wuzhi No. 2”. Vegetables.

[36] Guo H.B. (2009). Cultivation of lotus (*Nelumbo nucifera* Gaertn. ssp. *nucifera*) and its utilization in China. Genet. Resour. Crop Evol..

[37] Pyne M.E., Gold N.D., Martin V.J.J. (2023). Pathway elucidation and microbial synthesis of proaporphine and bis-benzylisoquinoline alkaloids from sacred lotus (*Nelumbo nucifera*). Metab. Eng..

[38] Wei X., Zhang M., Yang M., Ogutu C., Li J., Deng X. (2024). Lotus (*Nelumbo nucifera*) benzylisoquinoline alkaloids: Advances in chemical profiling, extraction methods, pharmacological activities, and biosynthetic elucidation. Veg. Res..

[39] Zhao X., Shen J., Chang K.J., Kim S.H. (2014). Comparative analysis of antioxidant activity and functional components of the ethanol extract of lotus (*Nelumbo nucifera*) from various growing regions. J. Agric. Food Chem..

[40] Estivi L., Buratti S., Fusi D., Benedetti S., Rodríguez G., Brandolini A., Hidalgo A. (2022). Alkaloid content and taste profile assessed by electronic tongue of *Lupinus albus* seeds debittered by different methods. J. Food Compos. Anal..

