# Supplementary material for: Comparative Analysis of Physicochemical Properties, Volatile and Metabolite Profile of Lotus Sprouts from Varying Cultivars and Grades
Source: Foods. 2026 Nov 4;15(21):3965. doi: 10.3390/foods15213965 (PMC13763200; doi:10.3390/foods15213965)
Supplement: Supplementary file 1 [file foods-15-03965-s001.zip › Figure S1.pdf]

a

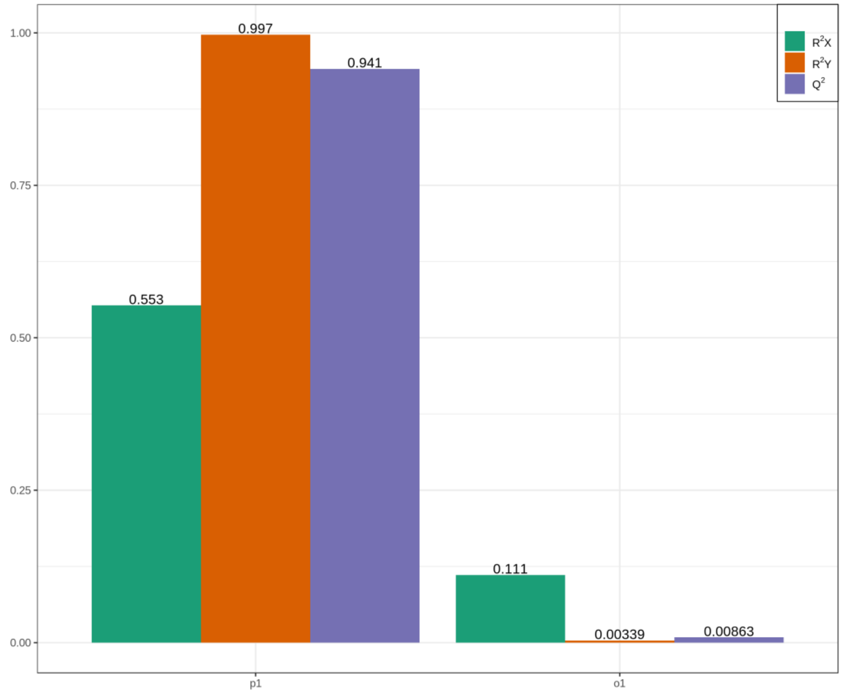

0.997
0.941
0.553
0.111
0.00339
0.00863
1.00
0.75
0.50
0.25
0.00
p1
o1
R2X
R2Y
Q2

b

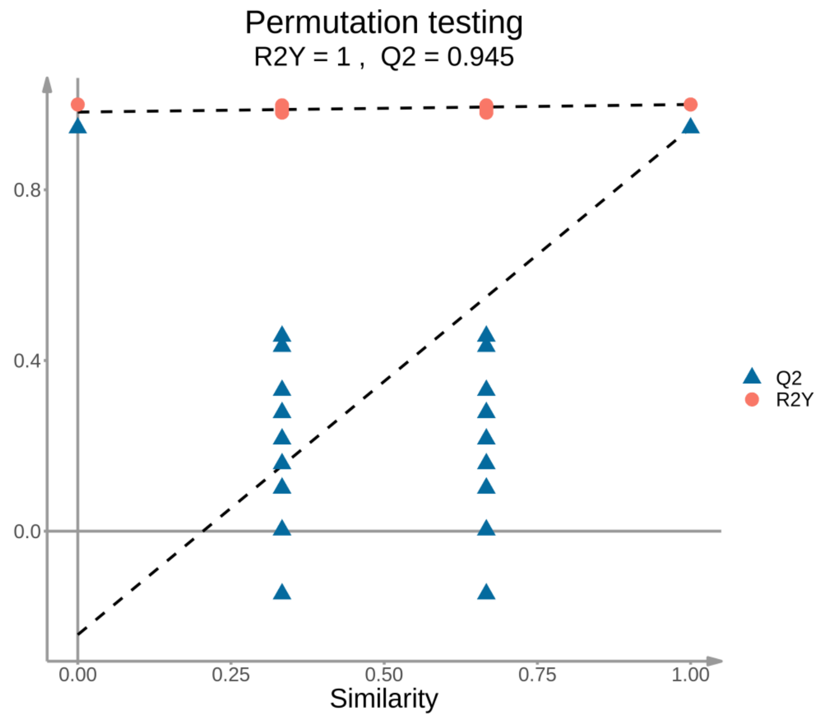

Permutation testing
R2Y = 1 , Q2 = 0.945
0.8
0.4
0.0
0.00
0.25
0.50
0.75
1.00
Similarity
Q2
R2Y

c

d

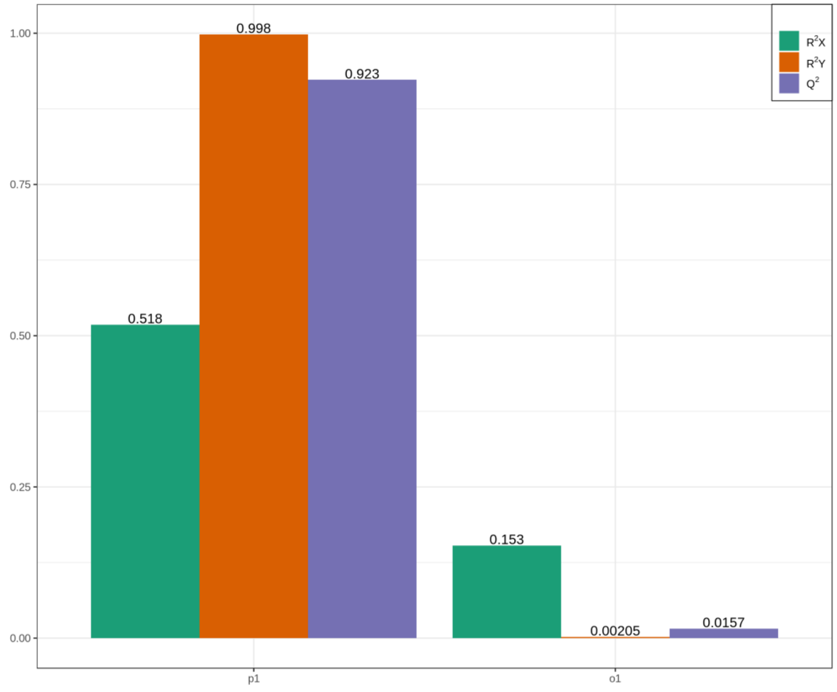

1.00
0.75
0.50
0.25
0.00
0.998
0.923
0.518
0.153
0.00205
0.0157
R^2X
R^2Y
Q^2
p1
o1

e

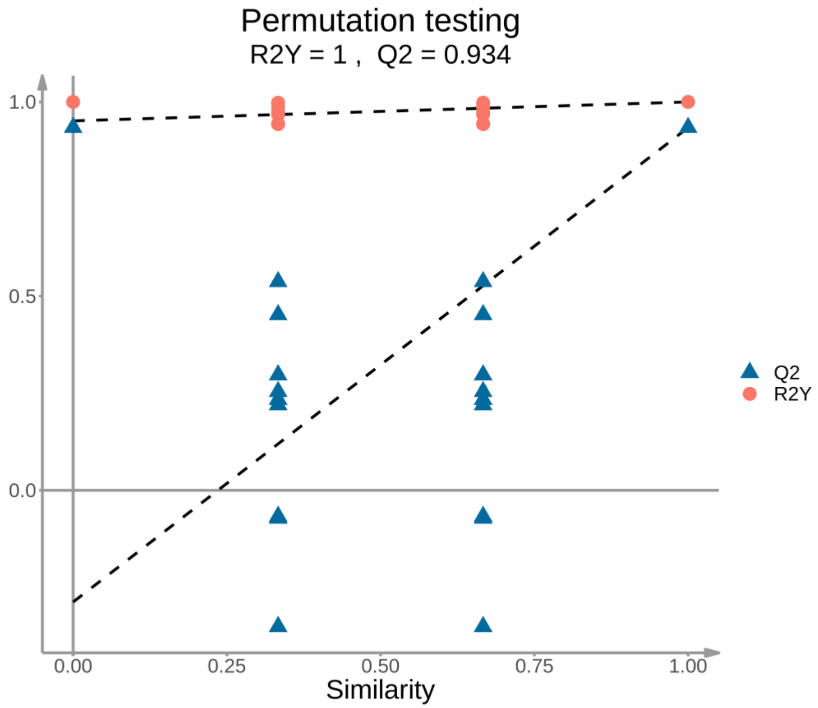

Permutation testing
R2Y = 1 , Q2 = 0.934
1.0
0.5
0.0
0.00
0.25
0.50
0.75
1.00
Similarity
Q2
R2Y

f

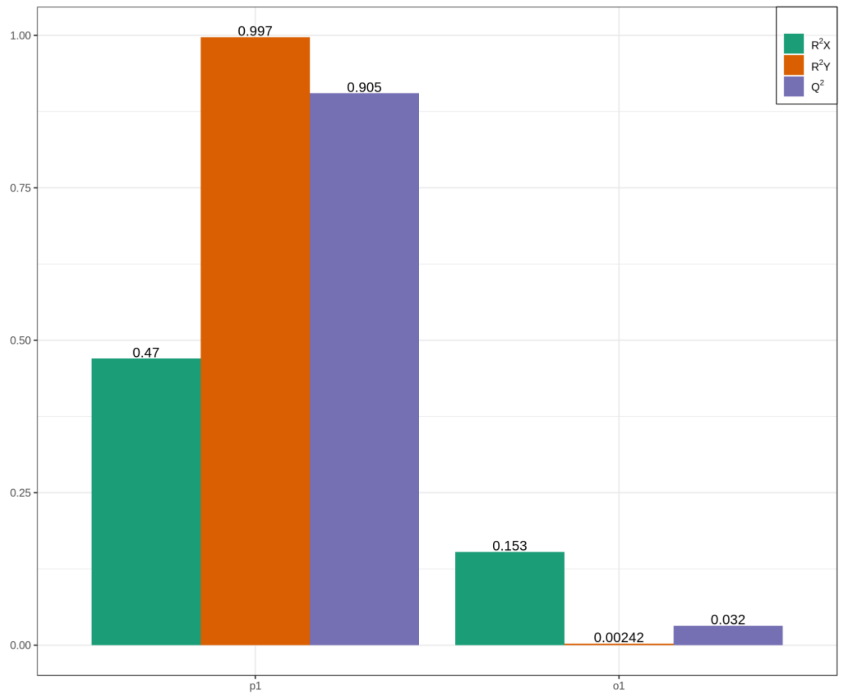

1.00
0.75
0.50
0.25
0.00
0.997
0.905
0.47
0.153
0.00242
0.032
R^2X
R^2Y
Q^2
p1
o1

g

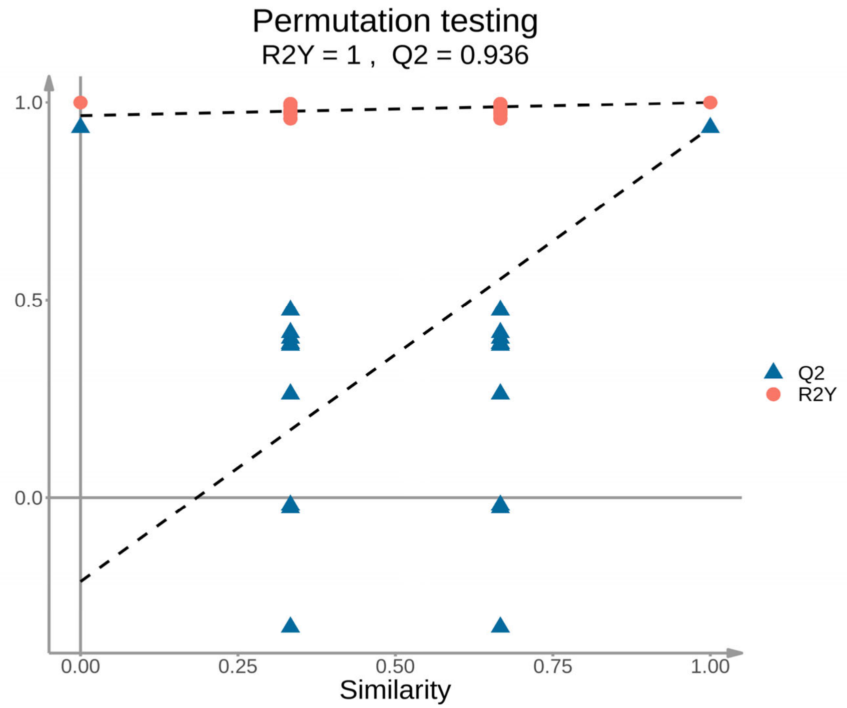

Permutation testing
R2Y = 1 , Q2 = 0.936
1.0
0.5
0.0
0.00
0.25
0.50
0.75
1.00
Similarity
Q2
R2Y

h

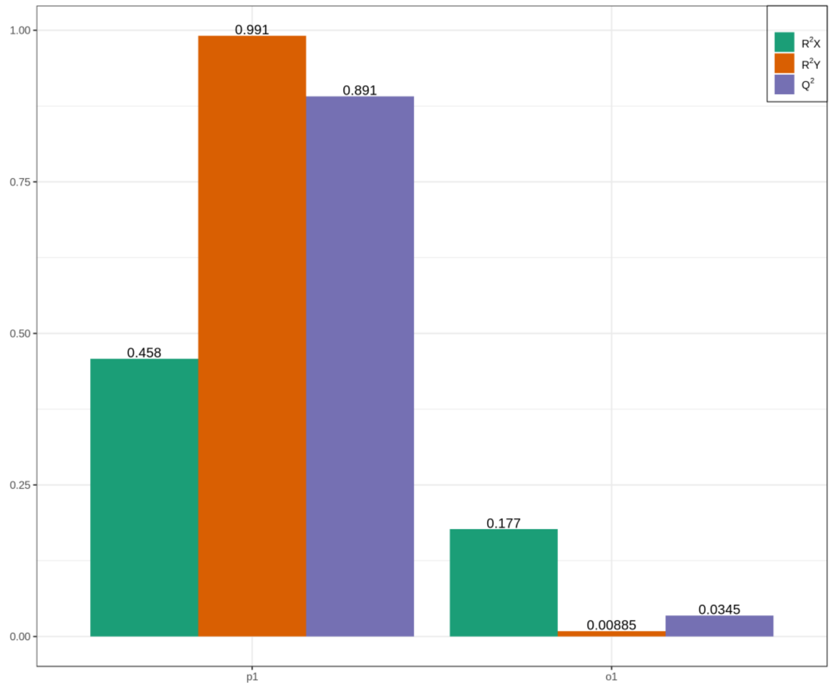

1.00
0.75
0.50
0.25
0.00
0.991
0.891
0.458
0.177
0.00885
0.0345
R2X
R2Y
Q2
p1
o1

i

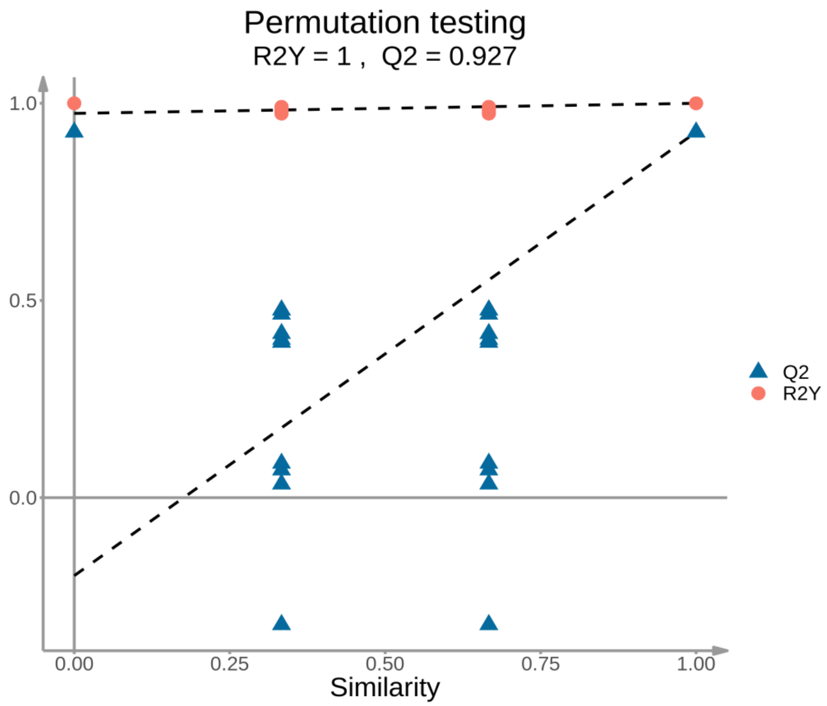

Permutation testing
R2Y = 1 , Q2 = 0.927
1.0
0.5
0.0
0.00
0.25
0.50
0.75
1.00
Similarity
Q2
R2Y

j

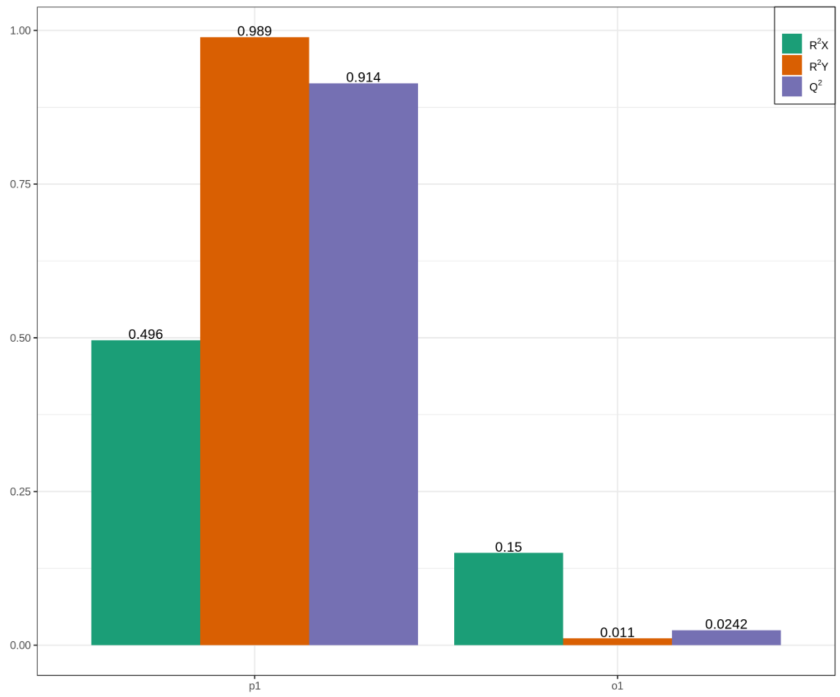

1.00
0.75
0.50
0.25
0.00
0.989
0.914
0.496
0.15
0.011
0.0242
p1
o1
R²X
R²Y
Q²

k

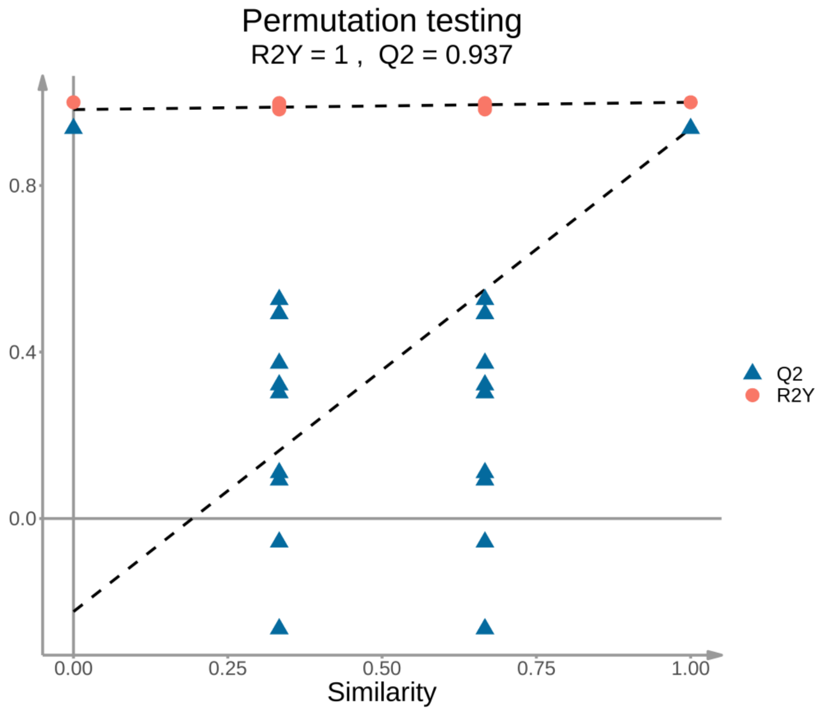

Permutation testing
R2Y = 1 , Q2 = 0.937
0.8
0.4
0.0
0.00
0.25
0.50
0.75
1.00
Similarity
Q2
R2Y

l

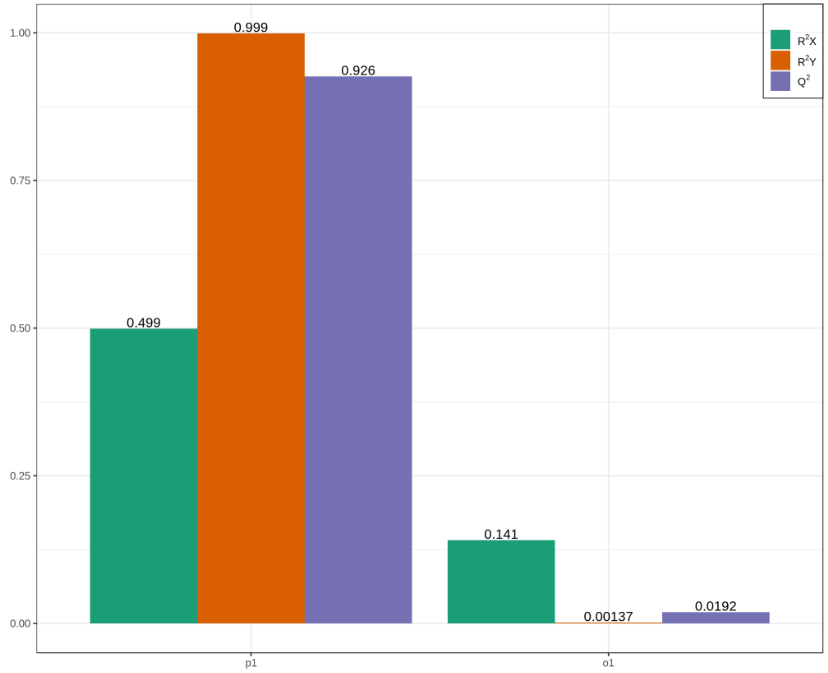
1.00
0.75
0.50
0.25
0.00
0.999
0.926
0.499
0.141
0.00137
0.0192
p1
o1
R^2X
R^2Y
Q^2

m

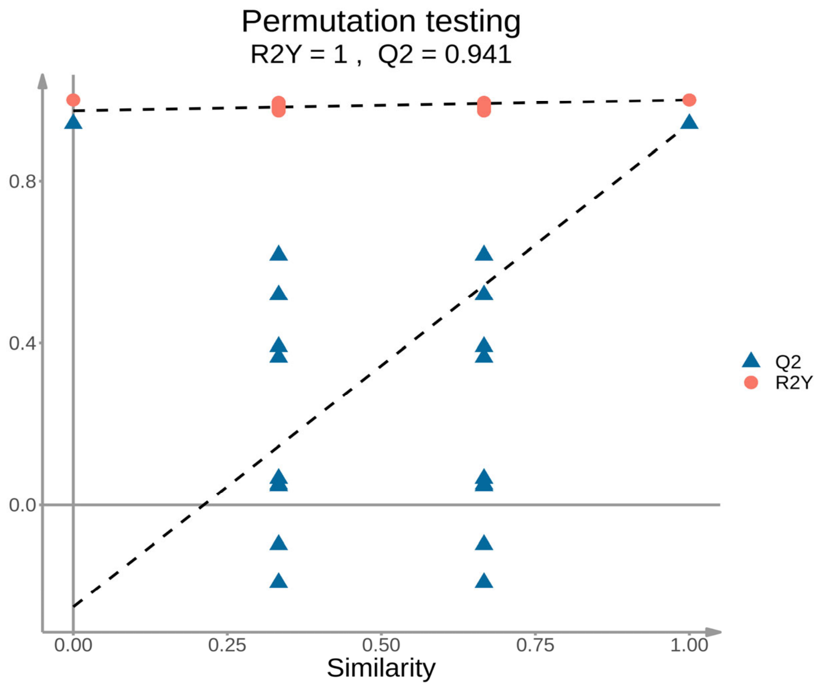
Permutation testing
R2Y = 1 , Q2 = 0.941
0.8
0.4
0.0
0.00
0.25
0.50
0.75
1.00
Similarity
Q2
R2Y

n

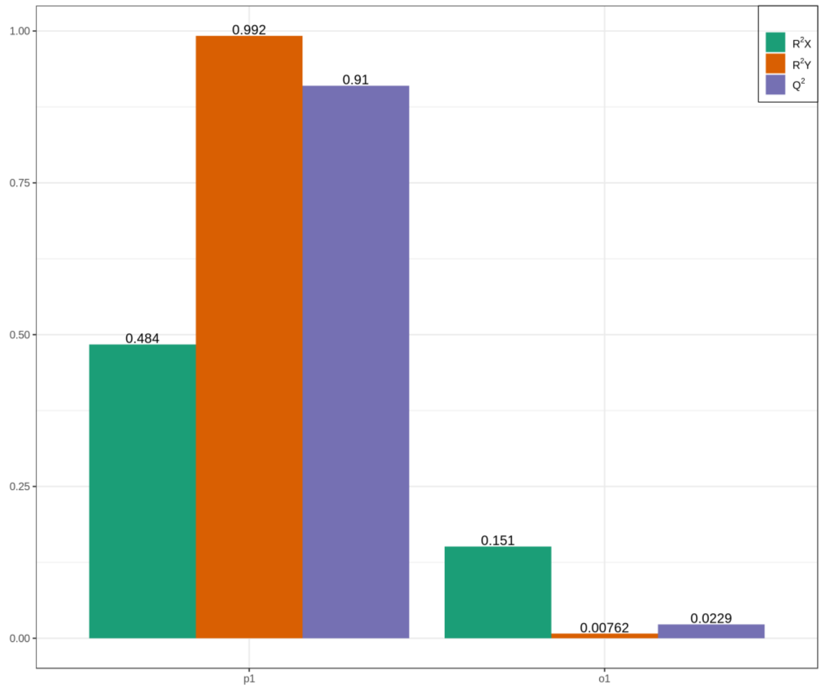

0.992
0.91
0.484
0.151
0.00762
0.0229
1.00
0.75
0.50
0.25
0.00
p1
o1
R2X
R2Y
Q2

o

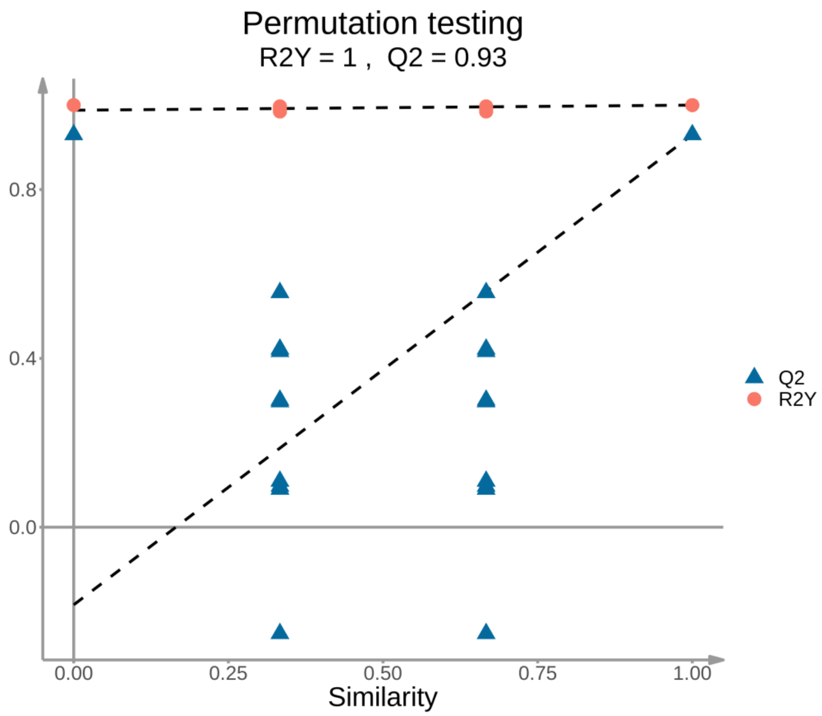

Permutation testing
R2Y = 1 , Q2 = 0.93
0.8
0.4
0.0
0.00
0.25
0.50
0.75
1.00
Similarity
Q2
R2Y

p

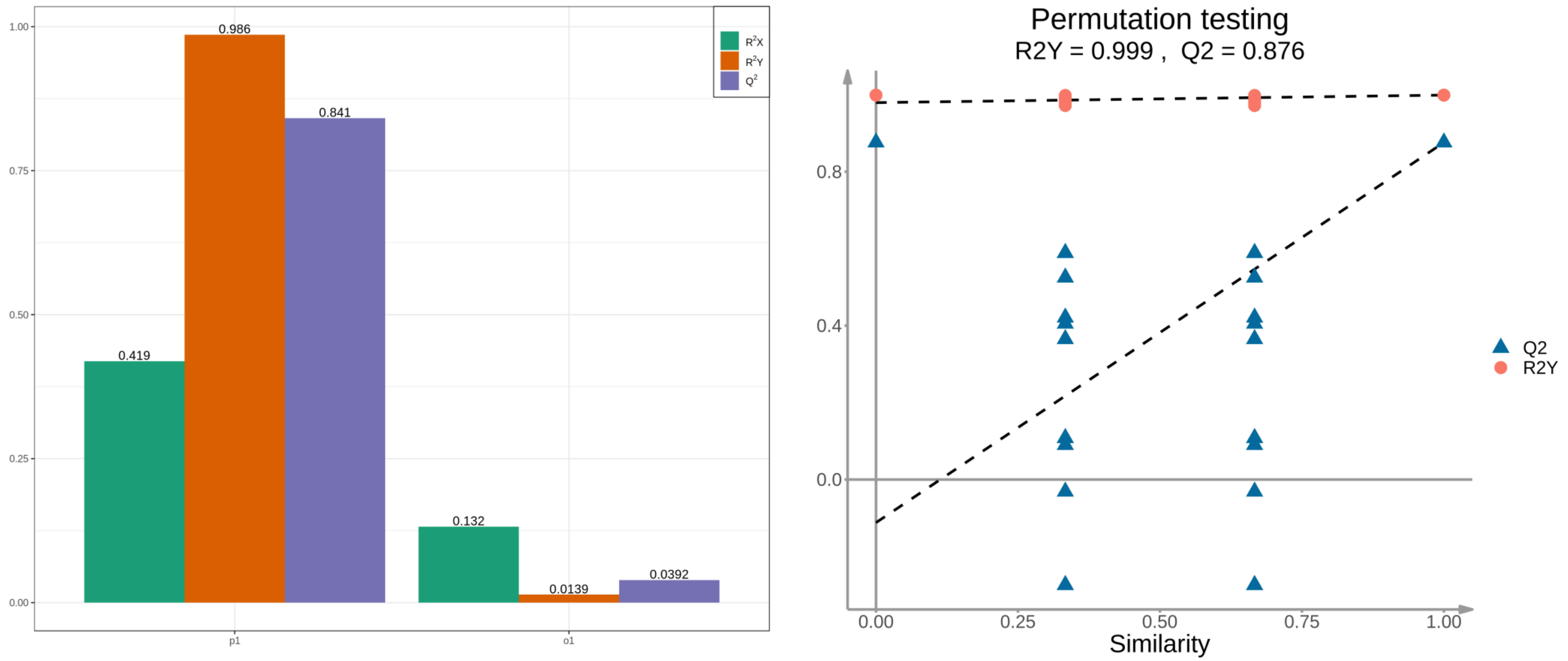

Figure S1. The OPLS-DA model parameter bar plots and 200-permutation test plots. (a,b) WZA1_vs_BYZ1A; (c,d) WZA1_vs_BYZ3A; (e,f) WZS1_vs_BYZ1S; (g,h) WZS1_vs_BYZ3S. (i,j) WZA1_vs_WZA2. (k,l) WZA1_vs_WZA3. (m,n) WZS1_vs_WZS2. (o,p) WZS1_vs_WZS3.
