# Supplementary material for: Comparative Analysis of Physicochemical Properties, Volatile and Metabolite Profile of Lotus Sprouts from Varying Cultivars and Grades
Source: Foods. 2026 Nov 4;15(21):3965. doi: 10.3390/foods15213965 (PMC13763200; doi:10.3390/foods15213965)
Supplement: Supplementary file 1 [file foods-15-03965-s001.zip › Table S1.pdf]

Table S1. Reference calibration curves and ion parameters.

Compounds	Calibration curve	R^2	Linearity range (μg/mL)	Quantifier ion (m/z)	Qualifer ions (m/z)
hexadecane	y = 1,204,734.584950x + 91,623.580553	0.9998	0.1-5	57	71, 85
1-octen-3-ol	y = 664,711.858460x - 123,662.329885	0.9987	0.1-5	57	82, 95, 110
2,4-di-tert-butylphenol	y = 237,035.911111x + 285,091.688889	0.9925	0.5-5	191	206, 192
benzothiazole	y = 1,459,162.480390x + 40,837.400397	0.9998	0.1-5	135	108, 90
