# Supplementary material for: Comparative Analysis of Physicochemical Properties, Volatile and Metabolite Profile of Lotus Sprouts from Varying Cultivars and Grades
Source: Foods. 2026 Nov 4;15(21):3965. doi: 10.3390/foods15213965 (PMC13763200; doi:10.3390/foods15213965)
Supplement: Supplementary file 1 [file foods-15-03965-s001.zip › Table S2-V1.pdf]

Table S2. Mass spectrometry MRM conditions.

Alkaloids	Parent ion (m/z)	Product ion (m/z)	Cone voltage (V)	Collision energy (ev)	Retention time (min)
Neferine	625.29	206.12*	18	32	0.72
		121.05		66	
Liensinine	611.27	206.12*	76	34	1.96
		107.01		54	
O-Nornuciferine	282.11	191.06*	42	26	2.15
		219.09		18	
Asimilobine	268.09	191.04*	36	24	2.26
		219.13		14	
Nuciferine	296.1	250.1*	32	20	2.33
		265.1		14	
Roemerine	280.09	249.08*	40	16	2.57
		191		32	
N-Nornuciferine	282.11	250.05*	28	18	2.59
		178.12		50	
Dehydronuciferine	294.11	279.01	24	16	3.8
		235.98*		30	

Note：* Denoting quantitative ions.
