# Supplementary material for: Comparative Analysis of Physicochemical Properties, Volatile and Metabolite Profile of Lotus Sprouts from Varying Cultivars and Grades
Source: Foods. 2026 Nov 4;15(21):3965. doi: 10.3390/foods15213965 (PMC13763200; doi:10.3390/foods15213965)
Supplement: Supplementary file 1 [file foods-15-03965-s001.zip › Table S3.pdf]

Table S3. Standards and reference calibration curves for alkaloids analysis .

Compounds	Purity	Calibration curve	R^2	Linearity range (μg/L)
Nuciferine	≥98%	y = 0.9239x + 7.7098	0.9958	10-500
O-Nornuciferine	≥95%	y = 0.8754x + 12.269	0.9916	10-500
N-Nornuciferine	≥98%	y = 0.9232x + 7.7161	0.9969	10-500
Neferine	≥98%	y = 0.9489x + 2.3232	0.9946	10-500
Dehydronuciferine	≥98%	y = 1.3831x + 11.94	0.9947	10-500
Asimilobine	≥98%	y = 0.8925x + 10.488	0.9941	10-500
Liensinine	≥98%	y = 0.9852x - 1.8109	0.9994	10-500
Roemerine	≥98%	y = 0.8737x + 13.447	0.9908	10-500
